# 3D printing processes in precise drug delivery for personalized medicine

**DOI:** 10.1088/1758-5090/ad3a14

**Published:** 2024-04-17

**Authors:** Haisheng Peng, Bo Han, Tianjian Tong, Xin Jin, Yanbo Peng, Meitong Guo, Bian Li, Jiaxin Ding, Qingfei Kong, Qun Wang

**Affiliations:** 1 Department of Pharmacology, Medical College, University of Shaoxing, Shaoxing, People’s Republic of China; 2 Department of Pharmacy, Daqing Branch, Harbin Medical University, Daqing, People’s Republic of China; 3 Department of Chemical and Biological Engineering, Iowa State University, Ames, IA 50011, United States of America; 4 Department of Pharmaceutical Engineering, China Pharmaceutical University, 639 Longmian Rd, Nanjing 211198, People’s Republic of China; 5 Department of Neurobiology, Harbin Medical University, Heilongjiang Provincial Key Laboratory of Neurobiology, Harbin, Heilongjiang 150086, People’s Republic of China; 6 These authors contributed equally.

**Keywords:** personalized medicine, 3D printing, dosage form, precise drug delivery

## Abstract

With the advent of personalized medicine, the drug delivery system will be changed significantly. The development of personalized medicine needs the support of many technologies, among which three-dimensional printing (3DP) technology is a novel formulation-preparing process that creates 3D objects by depositing printing materials layer-by-layer based on the computer-aided design method. Compared with traditional pharmaceutical processes, 3DP produces complex drug combinations, personalized dosage, and flexible shape and structure of dosage forms (DFs) on demand. In the future, personalized 3DP drugs may supplement and even replace their traditional counterpart. We systematically introduce the applications of 3DP technologies in the pharmaceutical industry and summarize the virtues and shortcomings of each technique. The release behaviors and control mechanisms of the pharmaceutical DFs with desired structures are also analyzed. Finally, the benefits, challenges, and prospects of 3DP technology to the pharmaceutical industry are discussed.

## 1. Introduction

Drug delivery is a process or method by which a drug is administered to an animal or person to achieve a desired therapeutic effect. A drug delivery system (DDS) is a device or formulation that delivers active pharmaceutical agents (APIs) into the body. It enhances their efficacy and safety by tuning the drug release rate, time, and location. The ultimate goal of DDS is to realize predefined drug release patterns that ensure optimized drug absorption and tissue distribution to improve clinical outcomes and patient compliance by providing safer, more effective, and more convenient delivery modes [1]. Drugs can enter the body through various routes using gastrointestinal, parenteral, transmucosal, transdermal, and pulmonary DDSs. They can produce systemic effects or realize target delivery to specific organs or diseases. The main factors in choosing the route of drug delivery are disease, the expected effects, and available medicine. Traditional formulations follow a ‘one-size-fits-all’ principle, which means adapting the patient to the drug rather than the other way around, resulting in a greater probability of side effects, most of which are dose-dependent and occur at therapeutic doses [2]. The traditional formulations can not achieve precise drug delivery according to patients’ unique requirements, especially for children and the aged, which may lead to poor patient compliance [3, 4]. Therefore, due to the spates of adverse reactions associated with ‘standard’ drugs, customized drugs and dosages have received increasing attention [5]. At the beginning of the era of personalized medicine in medical practice, patients will be guided to select the most effective and safest therapeutic regimen based on the results of genetic tests and proteomics and metabolomics analysis [6].

Personalized medicine will direct patients to take the right medicine in the correct dose and schedule [7–11]. Scientists have been exploring novel strategies to develop personalized medicine, including molecular diagnostics, pharmacogenomics, genetic profiling, artificial intelligence (AI), and three-dimensional printing (3DP) technology [12, 13]. Here, we have summarized the commonly used 3DP drug delivery technologies from the perspective of pharmaceutical engineering. We also review the production process of various dosage forms (DFs) through 3DP technology (table 1), discuss the challenges faced by this technology in drug delivery, and finally list its future development trends in the pharmaceutical field.

## 2. 3D printing process for drug delivery

**Figure 1.**
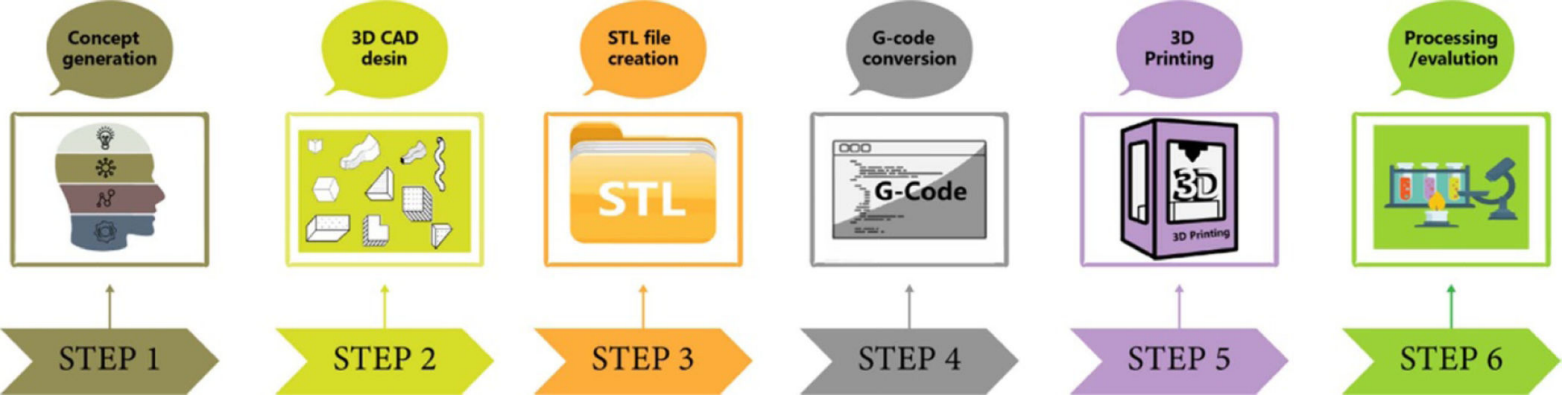
The basic 3D printing process includes six steps: concept generation, 3D CAD design, STL file creation, G-code conversion, 3D printing, and processing/evaluation.

3DP, called additive manufacturing, is a vital part of the rapid prototyping and manufacturing (RPM) technology family. In light of the category rules of the American Society for Testing and Materials (ASTM)/International Organization for Standardization (ISO), 3DP technologies are divided into seven families such as material extrusion, powder bed fusion, vat photopolymerization, binder jetting, material jetting, sheet lamination, and directed energy deposition [64]. Using decentralized accumulation molding technology, a complex 3D physical part is virtually cut into a series of digital two-dimensional (2D) layers according to a desired parameter. Then, these layers are stacked together. The digital computer-aided design (CAD) model guides the printing of any 3D object with a complex structure, combining discrete methods and manufacturing science. The final evolution of discretization is digital; the final goal is digital manufacturing [65]. RPM is implemented through computer-aided manufacturing, computer numerical control, and CAD. Together, these three technologies make it possible to print three-dimensional objects [66–68]. The 3DP process includes concept generation, 3D CAD design, stereolithography language (STL) file creation, G-code conversion, 3DP, and downstream subsequent processing and evaluation [66, 69]. The basic process of 3DP is shown in figure 1. In the past few years, 3DP has been applied in many fields, such as electronic products [70], tissue and organ regeneration engineering [71], food processing [72], medical equipment [73], etc. Because of the flexibility of 3DP technology, 3DP has been gradually applied in the pharmaceutical industry [74]. 3DP can generate customized complex geometric-shaped medications suitable for personalized treatment by adjusting drug size, dosage, shape, and release pattern, especially for children and elderly patients [75]. In the following sections, we will introduce the 3DP process for drug delivery.

### 2.1. Material extrusion system

Material extrusion system, also known as a nozzle-based deposition system, is a system that commonly integrates drugs, excipients, and adhesives through nozzles or holes and selectively allocates printing materials (ink) to manufacture objects based on the CAD-created 3D structure layer-by-layer. Based on the types of printing materials or inks used and their preparation methods, the extrusion systems are mainly divided into fused deposition modeling (FDM) and semi-solid extrusion (SSE) [76].

SSE is also called pressure-aided microsyringe (PAM). After heating or solid mixing, the initial semi-solid or low-meltingsolid mixing, the initial semi-solid or -point solid material is laden into the syringe as gel or paste with proper viscosity. The syringe is pushed with pneumatic, mechanical, or electromagnetic systems. Pneumatic-aided extrusion systems use pressurized air to extrude materials. The mechanical-based system with a pre-set piston or screw-drive device directly uses mechanical force to push the syringe to achieve material extrusion. The solenoid extrusion system uses electric pulses to make the valve at the bottom of the syringe open and print the gel or paste materials [77]. The diameter of the nozzle, nozzle stroke speed, extrusion speed, printing temperature, etc., are vital parameters that affect printing accuracy. Due to the use of paste or gel materials, further cooling and drying of the solidification process are required [78].

**Figure 2.**
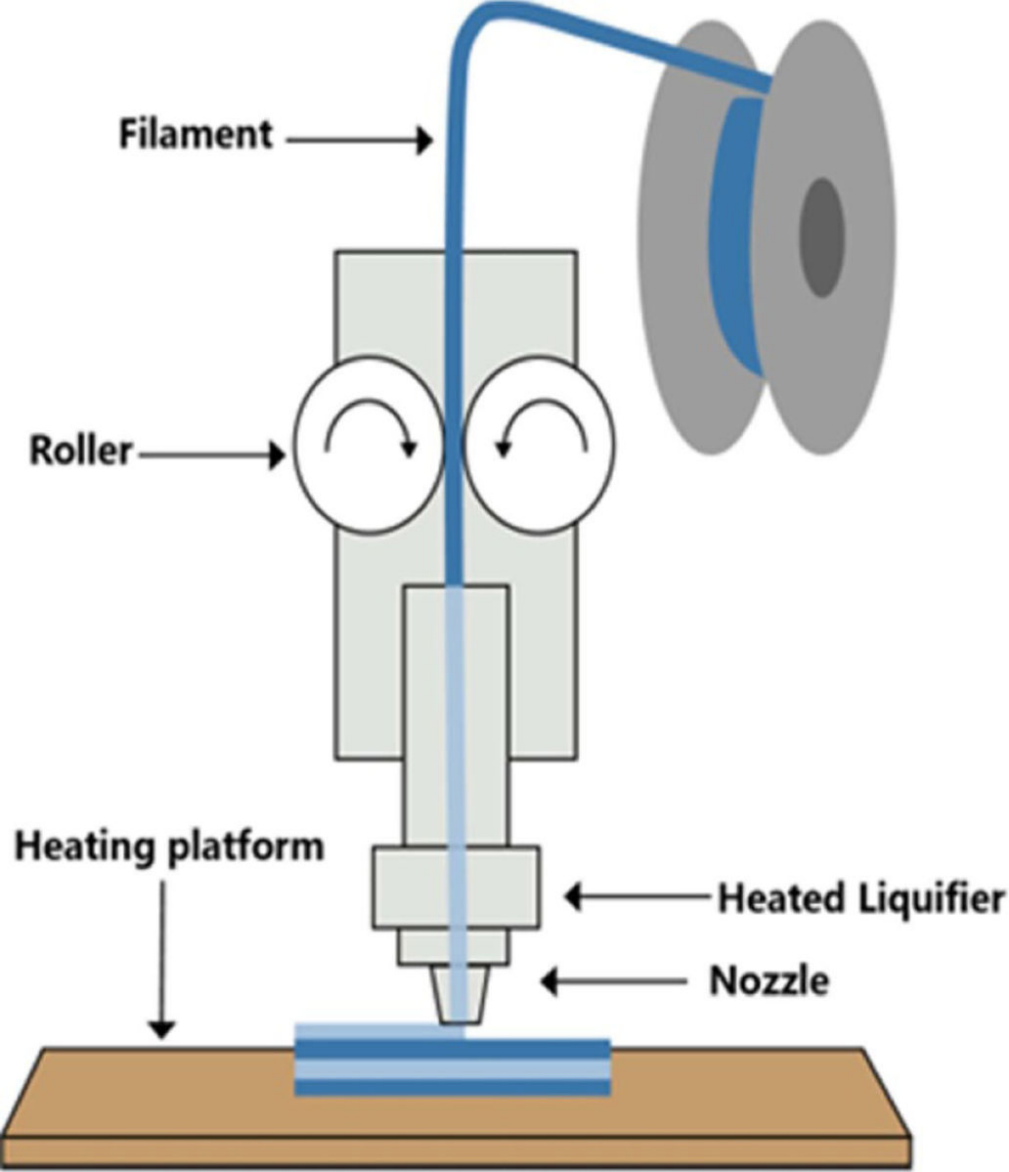
Schematic illustration of fused deposition modeling (FDM) 3D printer consisting of nozzle, heated liquefier, roller, heating platform, and rotary filament table.

FDM, also known as fused filament fabrication (FFF), is a combined process of printing thermoplastic materials and depositing them layer-by-layer on a heating platform (figure 2) [79]. At the beginning of FDM printing, thermoplastic polymer filaments are pushed by oppositely rotating clamping rollers into a heated liquidator whose temperature is set according to the type of filaments with different melting and glass transition temperatures (Tg) [80]. The polymer filament is molten at the printing temperature, while the solid part of the filament propels the fused polymeric materials through the printing nozzle [81]. The extruded molten polymer is then printed on a printing bed or heating platform according to the preconditioned coordinates [82]. In detail, the nozzle movement is governed by a three-axis system. The first layer is deposited on the *X-Y* plane following the CAD-designed path. After the first layer is completed, the nozzle moves up the thickness value of one layer along the *Z* direction to continuously print the next layer according to the set parameter in the software [83]. The deposited molten polymer immediately cools and cures. At the same time, the deposition and adhesion behaviors between layers are determined by the thermophysical properties, surface tension, melt viscosity, and melt flow behavior of the molten polymer [81, 84]. The process parameters that affect the printing performance are grating angle, layer thickness, bed temperature, extrusion rate, nozzle diameter, nozzle velocity, nozzle temperature, and so on; the correct selection of process parameters is the key to the success of FDM printing [85–94].

Three approaches are available to load APIs into 3D-printed products when printing drugs using an FDM printer. The first method called the ‘Impregnation—FDM,’ refers to loading drugs by passive diffusion. That is, FDM print filaments are soaked in the saturated solution of APIs for several hours to several days. After impregnation, the filaments are placed in an oven to dry until the weight is stabilized and kept in a vacuum desiccator [95]. Finally, the filaments loaded with APIs are raw materials for FDM printing [96]. The choice of solvent during impregnation is a crucial step for drug loading.

The solvent can dissolve the drug without altering the physical integrity and printability of the filaments [97–100]. The second approach is the ‘Print and Fill’ process [101, 102], in which 3DP prepares a hollow shell and then stuffed it with powder or liquid APIs [103, 104]. The shell is composed of thermoplastic polymer filaments, and the release of APIs is tuned by the dissolution of the ‘shell’ and the DFs in the shell [105]. The printing and filling steps can be simultaneous or sequential [106]. In major studies, the filling process was finished by hand, which was efficient when filling the API powder [107, 108]. The third method is a ‘combination of hot melt extrusion (HME) and FDM’ [109]. This method first uses the HME process to heat and soften a mixture of API and thermoplastic polymers. The molten material is then extruded through an extruder to produce APIs-loaded filaments [110]. The extruded filaments are then cooled, solidified, and used as raw materials for FDM printing [111]. The prescription and process parameters must be optimized during the HME process to obtain the best printable filament [112]. Each polymer/API mixture’s processing temperature must be reasonably controlled during extrusion. The processing temperature should be higher than the Tg of the mixture and lower than the degradation temperature, resulting in suitable melt viscosity and close interaction between APIs and polymer [113–115].

Recently, melt extrusion deposition (MED^™^) 3D is a new member of the extrusion technology family. This new technology allows the direct addition of raw materials of powder, which are transformed into softened/molten substances after heating and then precisely deposited layer-by-layer to print objects with the desired internal geometric structure. This technology overcomes the drawbacks of pre-manufacturing printable drug-loaded wire and improves the accuracy of printing printed objects [116].

### 2.2. Vat photopolymerization

Photopolymerization is a generic term used in many 3DP technologies. Photopolymerization is defined as the chain formation of polymer triggered by light. Light is only a starting tool that does not interfere with the propagation and termination of chain polymerization. It is the process from liquid to solid [117]. Photopolymer 3DP is a computer-controlled polymerization technology that produces concrete entities from a liquid resin by light exposure. Common photopolymer 3DP includes stereolithography apparatus (SLA), digital light processing (DLP), and continuous optical interface production (CLIP) [118]. The photopolymerization process comprises at least three parts: a light source, a photoinitiator (PI), and a photo-triggerable monomer/oligomer. Generally, the raw materials of photosensitive resin are composed of photo-triggerable monomer/oligomer and photoinitiator. The resin polymerization process can be divided into initiation, propagation and termination [119]. The kinetics of the curing reaction that occurs during polymerization is critical, affecting the curing time and the thickness of the polymerization layer. The reaction kinetics can be regulated by light source power, scanning speed, chemical composition, monomer amount, and specific photoinitiator (PI) [120].

#### 2.2.1. Stereolithography appearance

**Figure 3.**
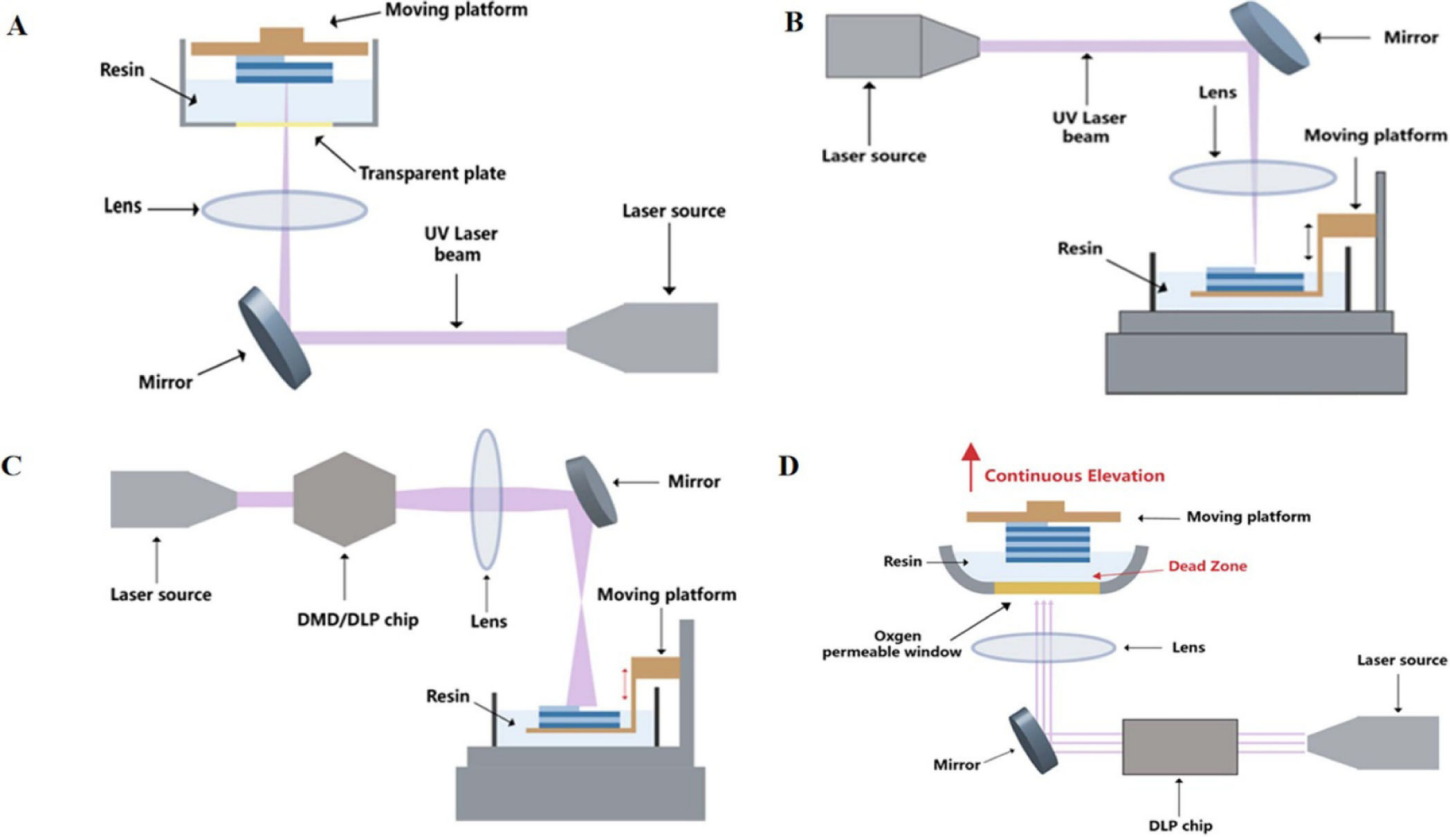
Schematic illustration of four vat photopolymerization 3D printing processes: (A) bottom-up stereolithography (SLA), (B) top-down SLA, (C) digital light processing (DLP), and (D) continuous liquid interface production (CLIP).

SLA creates 3D objects by UV laser-irradiated polymerization after the liquid resin is spatially maneuvered and solidified [121, 122]. The changes in printer structure bring two printing methods, namely top-down and bottom-up settings. In the setting of a bottom-up structure, the moving platform is at the upper of the printer while the light source (as the laser) is at the underpart, whereas in the setting of a top-down structure, the platform is at the underpart of the printer and the laser is at the upper [123]. During SLA printing in a bottom-up manner, after cured, the platform is lowered. A new layer of unpolymerized resin is printed on the surface of the polymerized materials to form the next cured layer (figure 3(A)). Another method cures the resin layer using a lucent plate as the bottom tray filled the resin, underneath which is equipped with a light source. Once the resin is cured, the platform ascends, and the unpolymerized resin redistributes the room between the sheet and the platform, allowing subsequent layers to cure on the polymerized surface in a top-down manner. This process is rehearsed until the object is wholly printed [124] (figure 3(B)). The resulting object is cleansed to remove excess resin. After curing, a UV oven can be used to solidify the object further and enhance its mechanical features [125]. Stereolithography can also be divided into two classes, namely single-photon and multiphoton methods, which differ in photoexcitation and absorption [126].

#### 2.2.2. Digital light processing

DLP is a 3DP technology used to cross-link photosensitive polymer monomers layer by layer [127] (figure 3(C)). The light source significantly differs between DLP and SLA strategies. The light source of DLP is a digital projector, while SLA is a point laser. Printing objects with DLP technology is faster because the projector can expose the entire layer simultaneously, whereas printing objects by SLA with a laser needs to draw point by point [128]. DLP uses a projector to project a cross-sectional image of an object into a liquid resin with light-sensitive properties. DLP technology is the key to DLP 3DP to determine image formation and printing accuracy. The digital microscope device (DMD), also named the DLP chip, is a critical part of the DLP printer. It contains an array of two million interconnected miniature microscopes that can be independently shifted between on- and off-state. The filled resin exposed to light is cured instantly by projecting a 2D light pixel onto a lucent plate. Construction time is highly reduced because it relies only on the filled resin’s deepness and the required exposure time [121, 129]. Compared with SLA, DLP 3DP has a higher printing resolution. But because of its limited projection size, only small models can be printed. Because the semiconductor packaging material does not tolerate ultraviolet light, a 405 nm LED lamp is used as the light source of the DLP printer. DLP 3DP usually uses free radical photosensitive resin as the printing material [129].

#### 2.2.3. Continuous liquid interface production

Essentially, CLIP technology improves SLA and DLP [129] (figure 3(D)). The key to the technology is adding a breathable window for oxygen and transparency for light accordingly. The window allows continuous printing by inhibiting free radical polymerization in the resin. In detail, during CLIP printing, a dynamic light projection (DLP) chip is used to repeatedly project the light pattern of each sheet into a photosensitive liquid resin through this unique window. Photoinitiators in the resin generate free radicals when exposed to light, triggering photopolymerization. However, when oxygen diffuses into the resin through the window, it acts as a free radical scavenger due to rapidly forming low-energy hydrogen peroxide radicals to inhibit the polymeric reaction. The polymerization inhibition in the resin close to the window forms light-curing ‘dead zones,’ a thin liquid layer between the window and the solidified surface of the parts. In this dead zone, solidification will not occur due to the diffused oxygen. With the increased distance from the window, oxygen concentration in the resin gradually reduces and curing light will not work in the area containing oxygen. Above the dead zone, the cured part is continuously drawn out of the resin bath, which suctions and constantly updates the active photosensitive resin to achieve continuous printing [130, 131].

During CLIP printing, the unique oxygen-inhibited dead zone realizes a constant and fast construction process. Theoretically, this change increases the potential to 1000 times faster than conventional 3D printers, whereas practically, to 25–100 times faster. It also eliminates the mechanical layering and coating steps that are repeated layer-by-layer in 3DP technology. The benefit of removing the layering step is that the physical forces exerted on the structure during construction are minimized, making it possible to create finer structures [131]. Theoretically, the same photosensitive resins used for DLP 3DP can also be used for CLIP 3DP. Although the CLIP process requires a higher viscosity of the material, especially in the fast printing process, low-viscosity resins with good fluidity are more suitable for CLIP printing [129].

### 2.3. Powder bed fusion

Powder bed fusion (PBF) is a process of selectively fusing powder particles into three-dimensional objects using thermal energy (such as laser). Currently, PBF includes four subset techniques, namely, selective laser sintering (SLS), selective laser melting (SLM), multi-jet fusion (MJF)/direct metal laser sintering (DMLS), and electron beam melting (EBM) [132].

**Figure 4.**
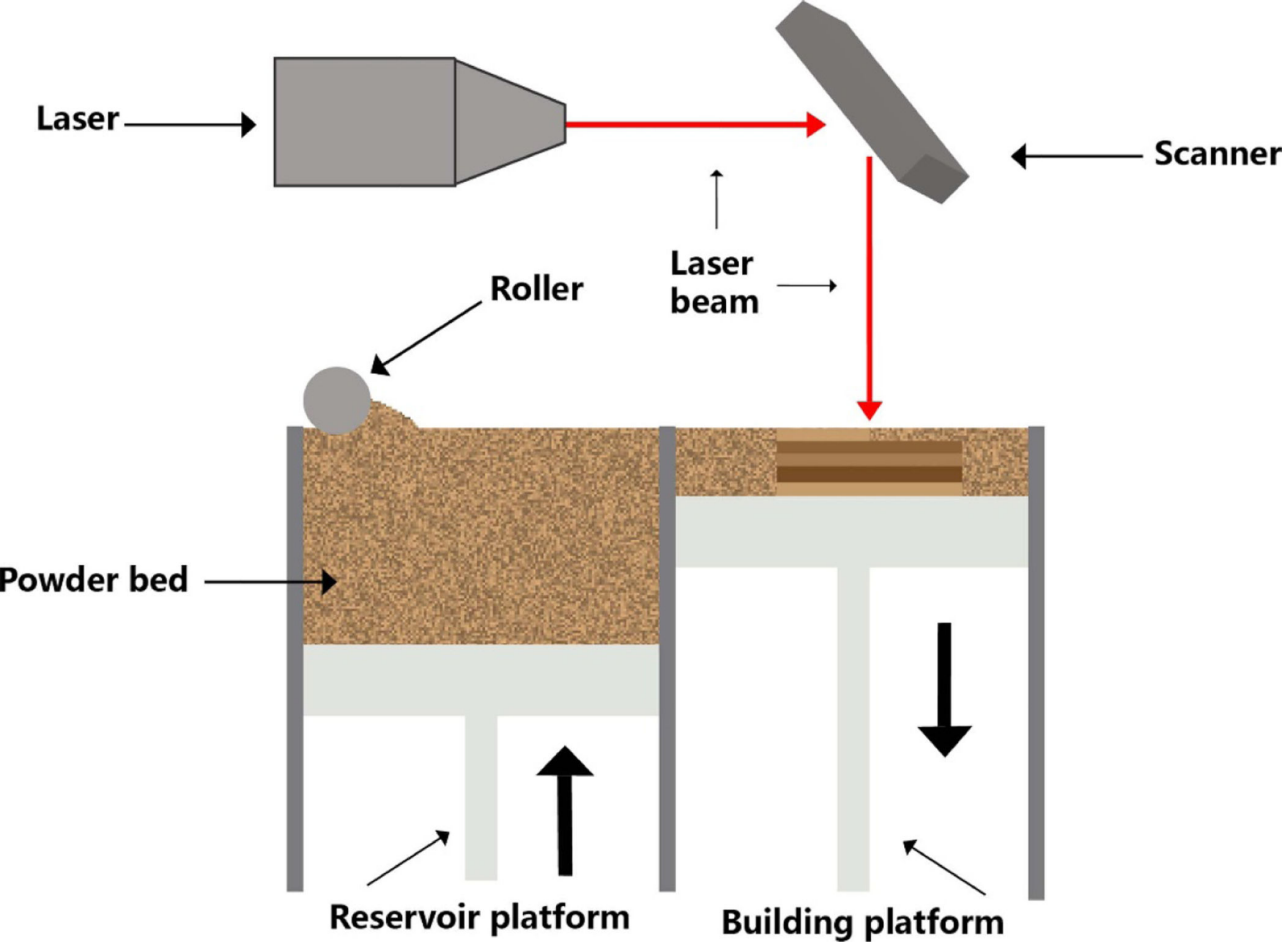
Schematic illustration of selective laser sintering (SLS), which is a subtechnique of powder bed fusion 3D printing.

SLS is a powder-based process that a high-energy laser is used to form a specific preconfigured structure on the surface of a powder bed and then sinter the powder particles together [133, 134] (figure 4). The SLS system has three main parts including the spreading powder unit, the powder bed, and the laser unit (laser and scanner). The printing process consists of a feeder spreading the powder onto the building platform and then elevating the platform to the topmost position. After that, a scraper, a roller, or a combination moves across the surface to flatten the powder. Next, the laser beam is activated, and the 3D part will be processed from the multiply-decomposed planes. Each plane has many laser-scanned basic units named vectors. The layer-scanned pattern and the direction and distance between the vectors are preset according to the 3D model. The laser then irradiates the surface of the powder following this pattern and site-specifically melts the exposure powder. The powder temperature is raised to one enough (but below the melting point) and forms the melted bridge among particles due to laser sintering. These powders solidify quickly after the laser leaves. The printing platform creates adequate space to spread a new powder layer while the powder reservoir system rises and the drum distributes a new powder layer. This printing is repeated until the object is finished. In the process, the unsintered raw powder, after each step, fills the space in the manufacturing room and supports the built parts, which are finally recycled [135, 136]. Therefore, since the unsintered powder particles remain loose and can be reused, which minimizes the waste of materials and promotes the recycling of raw materials [137], the processing parameters used in the printing process can statistically affect the features of the finished product. Thus, to obtain the best characteristics of the final product, the majorization of parameters must be performed to adapt to the properties of the powder and the expected applications. The key is to clearly understand the crosstalk of process parameters and their effects on the powder [138]. In the pharmaceutical industry, powders mainly comprise a mixture of thermoplastic polymers and API. Unlike traditional powder-compression methods, SLS prepares a 3D-printed object with loosely powder particles, resulting in a high-pore-filled matrix or a rapidly dissolved tablet. As a result, once dispersed in the solvent, the solvent molecules quickly penetrate the tablets, causing them to disintegrate [139].

As a PBF family member, I believe SLS is the most extensively applied printing technology in the pharmaceutical industry, surpassing SLM, MJF/DMLS, and EBM. However, the application progress of the other three technologies in drug development is slow due to limited available materials, high-energy laser beams, and high temperatures during printing processes [140].

### 2.4. Jet printing

**Figure 5.**
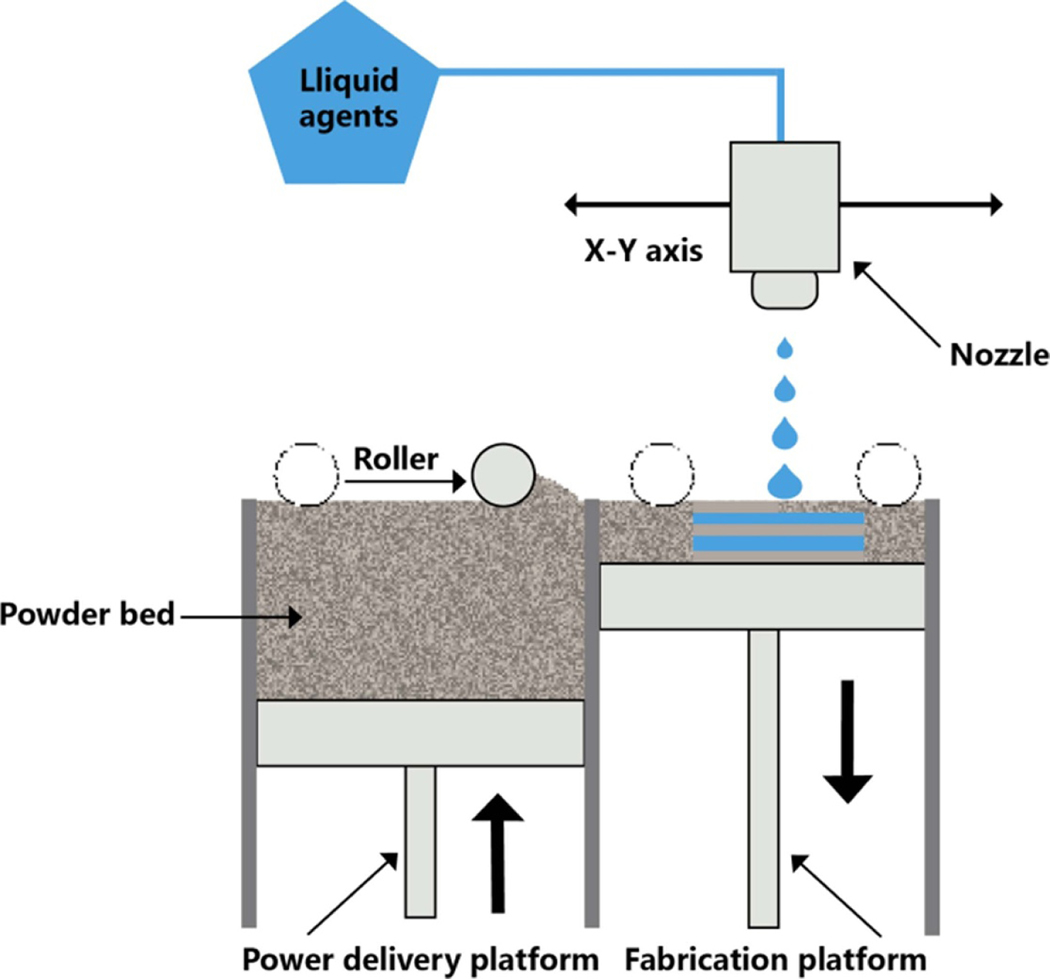
Schematic illustration of jet printing (JTP).

According to the ASTM/ISO standards, binder jetting and material jetting belong to additive manufacturing technology, which prints 3D objects based on jet printing mechanisms [141]. Jet printing (JTP) is a non-contact printing process in which tiny droplets are digitally controlled and dripped on a substrate by a pattern-generating device (figure 5). In the pharmaceutical industry, tiny droplets contain the appropriate drug mixture and the appropriate amount of excipients (called inks) are deposited layer by layer on the proper substrate [142]. JTP is a generic technology that can be applied to various fields. However, the printing principles are identical for all types of JTPs. This equipment mainly has a liquid agent and a powder bed. At the start of JTP, a thin-layer powder is spread on the constructed platform with the aid of a roller. The nozzle then precisely controls the released droplets of liquid adhesive onto the powder surface, bridging the powder particles. Once each layer is solidified, a new powder layer is coated on the constructed platform to print a new layer continuously [125]. In the binder jetting printers, the liquid agent is usually the adhesive solution, which is then cured by evaporating the solvent or chemical reaction as required, ultimately resulting in the joining of powder particles.

In contrast, the liquid agent is usually a photopolymer with a material jetting printer, and the powder particles are solidified or cured in each layer. Each IJP printer has a different controlled mechanism of droplet injection, which is classified as a droplet-to-droplet or droplet-to-solid process, depending on the differences in that nozzle sprays the droplet onto the substrate. Briefly, if the droplets jetted by the nozzle interact with other droplets to solidify the material, it is called a droplet-to-droplet jet. On the other hand, if it jets droplets onto solid material, it is called a droplet-to-solid jet. Because it is difficult to apply the drop-to-drop process in the pharmaceutics industry, most of the research in this field adopts the drop-to-solid process [143].

Two standard methods for preparing drugs use jet printing: (1) Ink contains drugs: the liquid-containing API (with or without adhesive) is deposited on a bed of excipient powder. (2) The powder contains drugs: the binder solution is deposited on the powder bed mixed with drugs [144]. Jet printing technologies are categorized into two types according to the physical processes that generate droplets: drop-on-demand jet printing (DOD) and continuous jet printing (CJP) [145].

In the process of CJP, the ink droplet production is stable and is controlled by the piezoelectric crystal high-pressure pump vibrating the nozzle. The printer’s signal selectively charges the resulting droplets. The charged droplets are deflected into a tank for recycling, while uncharged droplets are ejected onto the substrate to form an image [146]. The droplets are produced continuously, and their trajectories vary with the charge applied. CJP printing systems have droplets about twice as big as the nozzle hole diameter [147]. Because the ink droplets are produced continuously, their printing speed is fast. The printing nozzle is also hard to clog, and the volatile ink will dry quickly. CJP is mainly used for high-speed graphics applications [148]. However, due to the continuous generation of ink droplets during continuous jet printing, ink may be wasted, and resolution may also be reduced. Meanwhile, printing materials are limited to those materials that can be charged. In addition, continuous jet printers are relatively expensive, and daily maintenance costs are also higher [146].

In the process of DOD, ink is extruded from the nozzle when needed, and each drop of output occurs quickly to provide feedback to a trigger signal. Typically, DOD print heads contain multiple nozzles (generally 100–1000 nozzles, whereas professional print heads may contain only one nozzle). Unlike the ink injection caused by external fluid pressure in the CJP printer, the driving ink energy in the DOD printer is from the inside of the print head close to each nozzle. The diameter of the drip hole of the DOD printer ranges from 10 to 50 mm, and the corresponding volume of each drop ranges from 1 to 70 pL. The diameter of the droplet is similar to the diameter of the nozzle that sprays it [104]. The droplet generation mechanism shows that DOD printers can be divided into piezoelectric, electrostatic, electrofluid, and thermal jet printers [149]. Thermal jet printers use a thermal driver to create droplets. They usually have a microresistor that directly contacts the printing ink. The temperature of the heater usually reaches 200 °C–300 °C. This heat causes the ink to form bubbles, creating pressure pulses and ejecting them from the nozzle [150]. For thermal jet printers, the droplet volume is controlled by the temperature gradient and current pulse frequency, influencing ink viscosity, the boiling surface heating rate, and the applied voltage [151]. The piezoelectric jet printing system has a piezoelectric transducer (PZT) driven by voltage pulses. Due to the inverse piezoelectric effect caused by the piezoelectric drive, pressure (acoustic) waves are generated and propagated in the ink channel, spraying ink at the acoustic frequency [152]. The piezoelectric printing head can control droplet size and spraying speed by changing the driving voltage. Because ink injection depends on pressure wave propagation, fluid viscosity is a main speed-limiting factor [153]. Electrostatic jet printing systems use the electric field between the device and the basilar plate, imposing a voltage between the plate and the ink chamber to squirt the droplets out [154]. Electrostatic jet printing has better biocompatibility because it avoids using heat to create droplets and, at the same time, has color printing capability, which is most suitable for tissue engineering [155].

Before printing the jet-printing ink solution, the electric fluid is placed in a sealed container with a conductive nozzle. When voltage is imposed between the nozzle and reverse conductive holder, ink at the top of the nozzle forms a spherical meniscus shape. Afterward, a set potential difference between the nozzle and the plate will generate an electrostatic field, which induces the change of fluid meniscus to a cone structure [156]. Finally, ink is ejected from the tip of the cone. The back pressure, the distance between the nozzle and the plate, and the imposed voltage will affect the droplet size and ejection frequency [157]. The droplet size generally decreases with the increased charged voltage, but the medium transport fails when the droplet size exceeds 400 *μ*m [158].

## 3. Pharmaceutical dosage forms by 3DP

**Figure 6.**
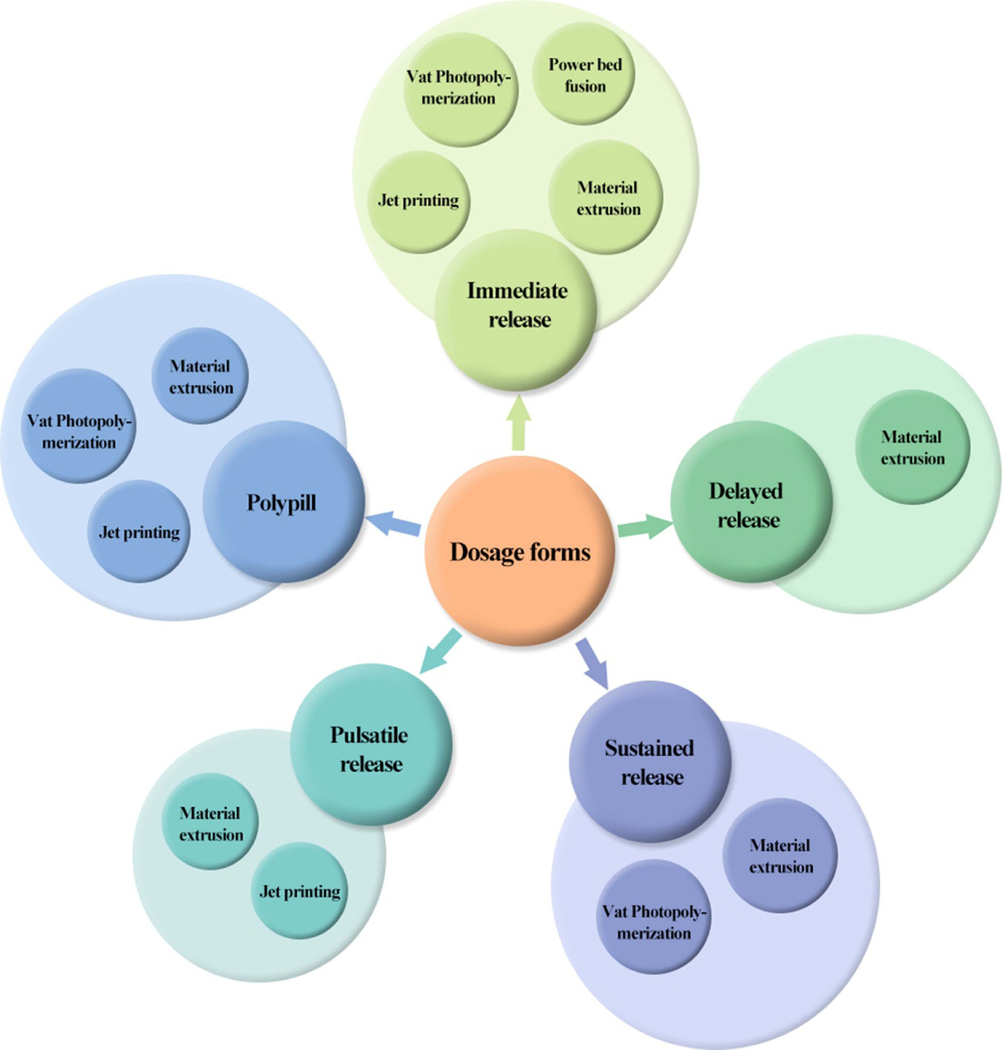
Diagram of different 3DP techniques applied to drug dosage forms.

To maximize the benefits and minimize reverse results of pharmaceutical DFs, 3DP technologies are used to design the specific formulation’s size, shape, and structures to realize the personalized treatment. According to the unique requirements of patients, the administration routine and release behavior are recognized based on the different DFs, including immediate release of dosage forms (IR-DFs), delayed release dosage forms (DR-DFs), sustained release of dosage forms (SR-DFs), pulsatile release of Dosage forms (PR-DFs), personalized combination and polypill, and microneedles (MNs) (figure 6).

### 3.1. Immediate release of dosage forms

IR-DFs refer to a large class of preparations that can quickly disintegrate or dissolve after administration. After disintegration or dissolution, the quickly-released drugs can be absorbed through the oral or gastrointestinal mucosa [159]. The most commonly-used oral medicines on the market today are tablets with immediate release (IR) behavior, which ratio is close to 80% of new drug entities (NDE). Conventional IR-DFs are usually obtained by compressing APIs with appropriate disintegrating excipients that allow the APIs’ rapid disintegration and timely dissolution [22].

The jet 3DP method has proven its great potential for producing IR-DFs. The 3DP method is different from the traditional compression of powder and integration mechanism of tablets. The printing tablets by 3DP use the interaction between powder and binder to achieve powder curing. Thus, a DF with low density, high porosity, and uniform dispersion of APIs and filling particles can be produced [160]. It is difficult to fabricate such highly porous structures using conventional tablet pressing without compromising the tablets’ mechanical strength and physical integrity. When introduced, a solvent can penetrate these structures very rapidly, resulting in rapid decomposition of tablets and IR of the API [161]. Based on the inherent characteristics of jet 3DP, it has been popularly used in the studies of IR tablets [14, 15, 162]. Currently, an instant 3D-printed levetiracetam (Spritam^®^) tablet for epilepsy is the only one officially agreed upon by the Food and Drug Administration (FDA), which Aprecia Pharmaceuticals has developed based on the inkjet printing technology (ZipDose). A single sip of the liquid can cause the tablet to break down quickly in 10 s. A single dose of the tablets is up to 1000 mg, which meets the needs of those with difficulty swallowing [163]. This is a milestone in using 3DP technology in the pharmaceutical industry and powerfully stimulates the development of 3DP in the pharmaceutical field.

**Figure 7.**
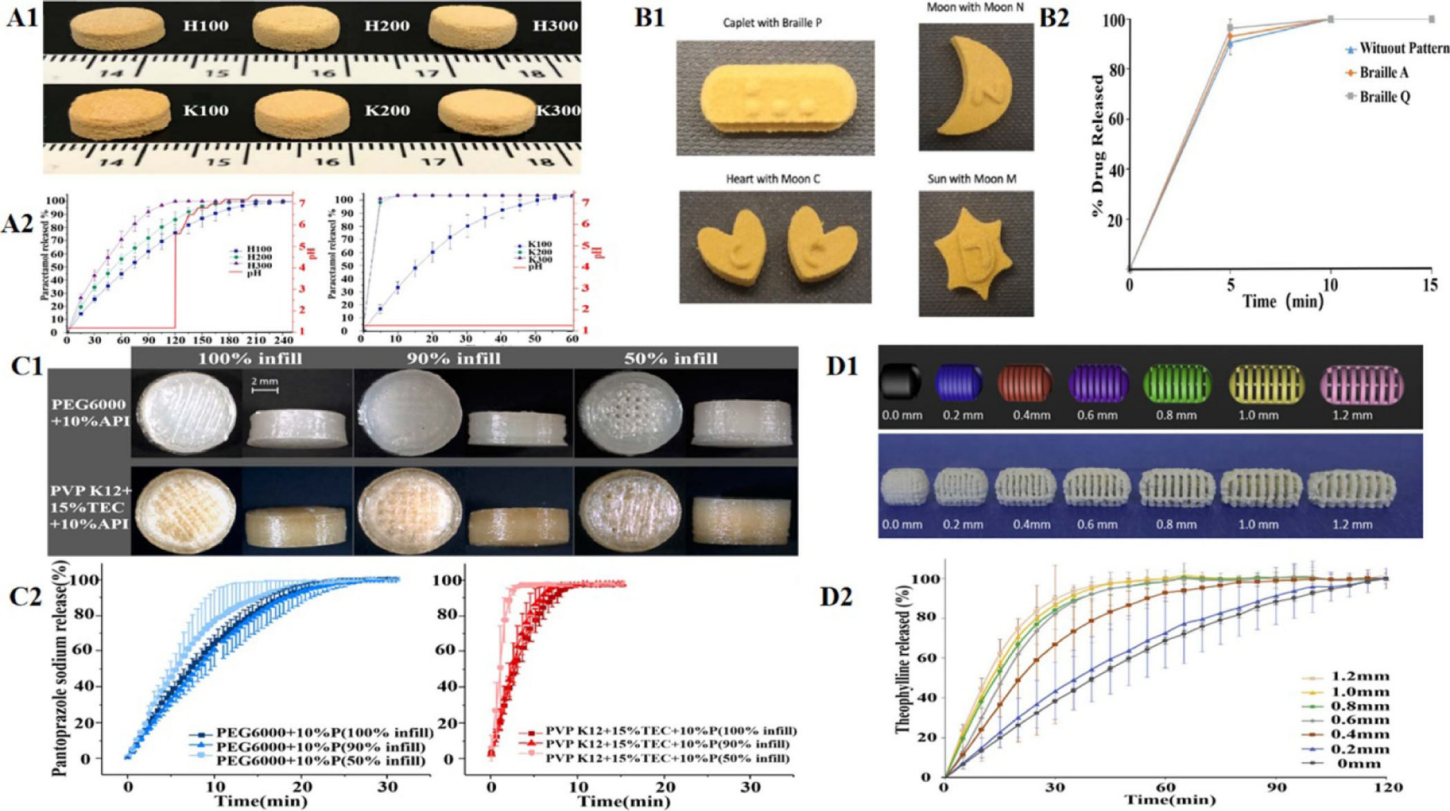
Examples of immediate release dosage forms (IR-DFs) of 3DP. (A1) Images of the HPMC and Kollidon printlets fabricated by the SLS printing process. Reprinted from [16], Copyright (2018), with permission from Elsevier. (A2) *In vitro* release curves of HPMC and Kollidon printlets. (B1) Printlets with various shapes, including Braille or Moon patterns, fabricated by the SLS printing process. Reproduced from [18]. CC BY 4.0. (B2) Drug dissolution profiles of the printlets. (C1) 3DP tablets with different filling ratios manufactured by the FDM printing process. Reproduced from [19], with permission from Springer Nature. (C2) *In vitro* release curves of 3DP tablets with varying loading ratios. (D1) Rendered and photographic images of tablets were fabricated by the FDM printing process. Reprinted from [21], Copyright (2018), with permission from Elsevier. (D2) *In vitro* release curves of 3D printed tablets.

For IR-DFs, porosity is crucial as it directly affects APIs’ disintegration process and bioavailability. Porosity determines the permeation of the environmental medium and the solvation of APIs to regulate drug release, especially for formulations without disintegrants. 3DP based on the SLS 3DP can also be used to make such porous solid DFs with IR properties [164]. Taking acetaminophen (5%) as a model drug, Fina *et al* selected the SLS 3DP method to compress cylindrical oral disintegrating tablets that contain vinylpyrrolidone-vinyl acetate copolymer (Kollidon VA 64), hydroxypropyl methylcellulose E5 (HPMC E5) as well as Candurin Gold Sheen (3%) as absorbance enhancers (figure 7(A)). The authors confirmed that accelerating the SLS laser’s scanning speed could reduce the material’s sintering effects and leave more gaps between granular materials, thus accelerating the dissolution of the tablet [16]. The interaction between process parameters and formula factors is a crucial quality attribute affecting APIs’ release behavior in the formulation. Used diclofenac sodium as a model drug and Kollidon VA 64 as particle material, lactose monohydrate and Candurin^®^ NXT Ruby Red as absorbance enhancers, Sogra *et al* prepared IR cylindrical tablets based on SLS 3DP technology. Box-Behnken response surface method (RSM) was utilized to screen the prescription of DFs. The optimized process parameters printed the tablets with ideal characteristics (good mechanical integrity, high disintegration/dissolution rate) [17].

Additionally, the interaction between formula ingredients and the uniformity of drug distribution are vital concerns during printing. SLS 3DP can significantly improve the treatment compliance of vulnerable populations such as children, the elderly, and disabled patients. For example, Awad *et al* used SLS 3DP technology to produce oral disintegrating tablets (ODTs) with braille and moon patterns. In this tablet, paracetamol was taken as a model drug, Kollidon VA64 as printing material, and Candurin^®^ Gold Sheen as absorbent (figure 7(B)). These researchers kindly created these personalized solid oral DFs for patients who are blind or visually impaired. This creation could improve medication independence and compliance and reduce errors in visually impaired individuals [18]. Although Candurin Gold^®^ Sheen is the most widely used sinter in SLS 3DP, explorations into novel sinter technologies are urgent because the common sinters in the DFs in the literature have reached the 3% safety limit (weight ratio). Zhang *et al* developed an oral anti-tuberculosis tablet (model drug, isoniazid) using carbonyl ferromagnetic particles as sinter materials by the SLS 3DP process. The data showed that carbonyl iron not only efficiently absorbed the laser energy, resulting in the glittering sintering of the tablets, but also improved the drug release under magnetic fields by utilizing its magnetic properties [165].

SSE is also an appropriate technology for producing IR tablets. During the treatment of pediatric epilepsy, levetiracetam dosage needs to be subsequently increased within a few weeks [166]. 3DP shows the flexibility to prepare tablets, making it easy to modify the dosage regimen that has been followed traditionally. The tablets released APIs within from 10 to 20 min after they met the release medium, which depended on the chosen excipients. Furthermore, organic solvents avoiding the printer nozzle blockage were not needed. In the following work, levetiracetam-loaded tablets with different printing layers were printed to meet the other requirements of pediatric needs. The layer of 3DP tablets determined the dissolution behavior of APIs, and the more layers, the slower the release. All tablets with different formulas disintegrated within 3 min, thus meeting the requirements of the European Pharmacopoeia [25].

Typically, the dissolution rate of tablets printed by FDM is relatively slower than that of ink-jet-printed IR tablets, probably due to the compactness of the used polymer and the cured molten materials. Therefore, to achieve IR behavior, the porosity of tablets should be increased by selecting the appropriate polymer and changing the filling rate of tablets during FDM printing [167]. Kempin *et al* used the heat-sensitive drug pantoprazole sodium as a model drug. They successfully prepared drug-loaded filaments with the medical-grade polymers polyethylene glycol 6000 (PEG 6000), polyvinylpyrrolidone (PVP K12), and 15% triethyl citrate (TEC) as plasticizers using a hot melt extruder (figure 7(C)). Afterward, FDM was used to print a cylindrical tablet with a 10% (w/w) loading capacity of pantoprazole sodium, a standard dose of 20 mg. To avoid the filaments’ breakage caused by the gear’s force during the extrusion process, the commercial polylactic acid (PLA) filaments are placed behind the drug-loading filaments to push them. In in vitro drug release experiments, the drug release times of PVP K12 tablets and PEG 6000 tablets were within 10 min and 29 min, respectively. When the filling rate of PVPK12 tablets was reduced to 50%, the total drug release time was changed from 10 min to an ultra-rapid three minutes [19]. However, pantoprazole sodium is unstable in an acidic environment. Unfortunately, they did not focus on the critical issue of acid resistance of drugs in the stomach. In addition, blank granules of excipient (for the studies of API loading, optimizing the ratio of polymer to disintegrant (key material properties, KMP)) in FDM-printing DFs were also successfully developed for IR tablet [168, 169].

Studying the effects of different components and their potential interactions on drug release patterns is equally essential for preparing IR-DFs using FDM. Than and Titapiwatanakun developed a combination of FDM printing and hot melt extrusion based on a Quality of Design (QoD) method. IR tablets were successfully printed without plasticizer in the polymer mixtures containing sodium starch glycolate (disintegrant), hydroxypropyl cellulose (HPC), Kollidon^®^VA 64, Eudragit^®^EPO, and theophylline contents (10%, 30%, or 60%, w/w) [20]. Using the FDM process to print tablets with complex geometric structures improved their in vitro release properties, leading to the successful development of IR tablets [170]. Arafat *et al* used theophylline as a model drug, HPC-SSL (full name) and triethyl acetate as excipients, and HME-FDM as a printing method to successfully print a capsule shape of the unique tablet with multiple internal gaps (figure 7(D)). Drug release experiments in vitro showed that forming a single large cellulose matrix could be prevented when the inner gap was ⩾1.0 mm. Rapid drug release is achieved by promoting erosion and maintaining the diffusion pathway at the lowest swollen level of each block [21]. Taken hydrochlorothiazide as a model drug and HCT: TCP: TEC: Eudragit E (37.5%: 12.5%: 3.25%: 46.75% wt) as the optimized prescription of excipients, Sadia *et al* also used the HME-FDM method to print capsule tablets with channels inside. This complex geometric structure increased the tablet’s surface area, helping solvent penetration into the tablet. When the diameter of channels ⩾0.6 mm, they are sufficient to meet the pharmacopeia requirement for IR of tablets. Using many shorter channels could more effectively accelerate drug release from tablets than more extended channels. The authors held that short-channel tablets work well because the short-channel reduced the flow resistance of the media in the dissolution test, leading to quicker disintegration of the tablets. In addition, the authors confirmed that the channel system was more suitable for the non-swelling materials because the expandable polymer matrix may cause the channel to be narrow or even close to prevent the introduction of the medium [22, 23]. Taken polyvinyl pyridine-vinyl acetate copolymer (Kollidon^®^VA64) and the mixture of Kollidon^®^VA64 and hydroxypropyl methylcellulose (Affinisol^™^15cP) as polymeric matrix and haloperidol as a model drug, Patel and Serajuddin chose glutaric acid as a super solvent to successfully print fast release tablets using HME-FDM method, following acid-base supersolution (ABS) principle. Because ABS technology has the advantages of accelerating drug release, enhancing drug-polymer miscibility, improving the printability of polymers, and decreasing the printing temperature, the combination of ABS technology and the HME-FDM method may make an overshoot in the studies of FDM 3DP tablets [24].

### 3.2. Delayed release dosage forms

DR-DFs are formulations that delay drug release until they pass through the stomach, preventing drugs from potentially irritating the gastric mucosa or being inactivated by gastric juice [171, 172] Conventionally, this is achieved by coating the core (e.g. tablet, pellet) with a 30–100 *μ*m thick film. When the pH value of the intestine reaches 5.5 or higher, the film is dissolved. However, this approach is complex for 3DP of DFs because it is impossible to construct fragile and sealed movies based on the current 3DP methods [173]. Two solutions to this problem are simultaneous 3DP of coat and core (fluid storage system) or 3DP of enteric matrix (monolithic system) [134, 174].

**Figure 8.**
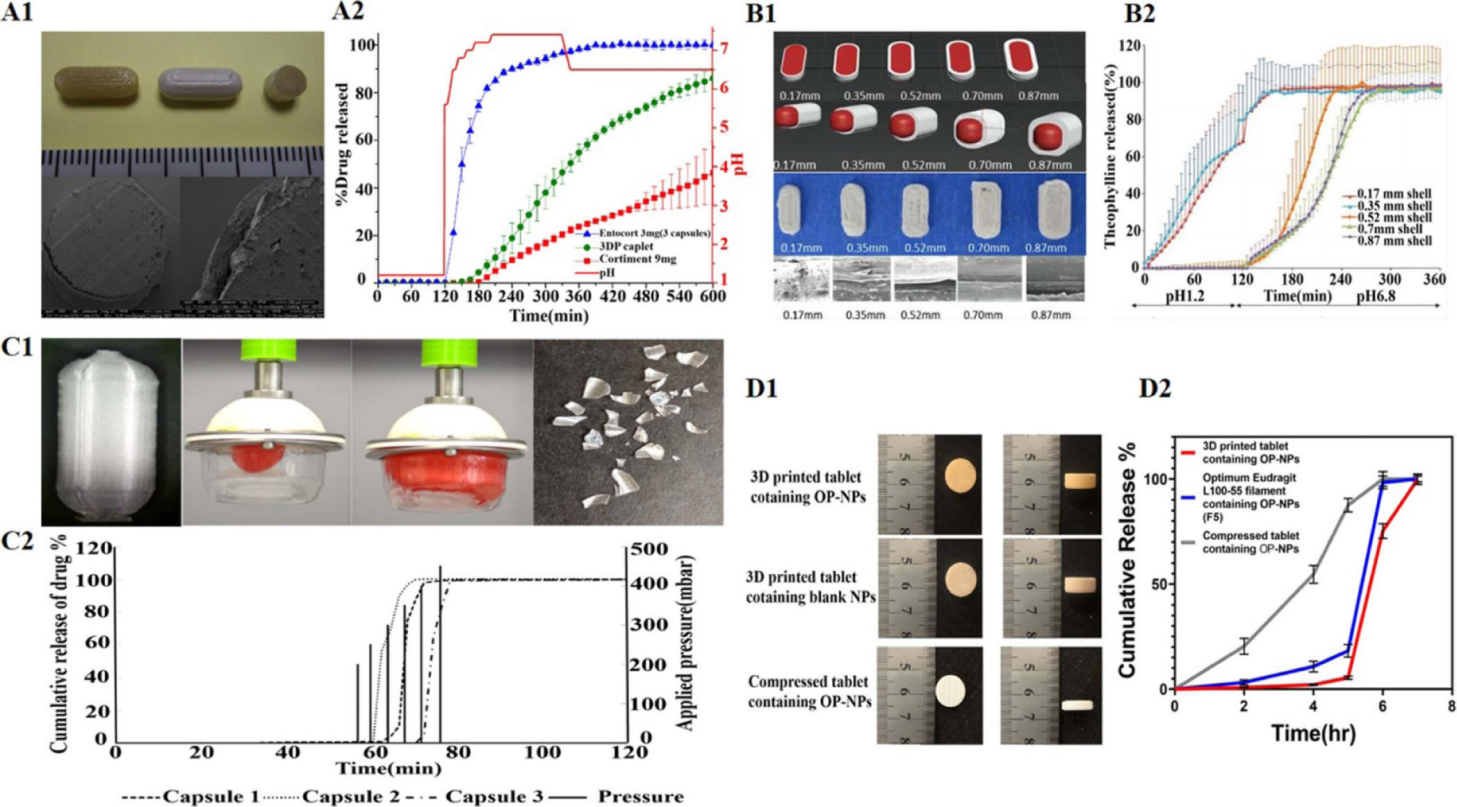
Examples of delayed release dosage forms (DR-DFs) of 3DP. (A1) Images of 3DP fabricated caplets prepared by the combined process of HME-FDM and fluidized bed. Reprinted from [26], Copyright (2015), with permission from Elsevier. (A2) Drug release curve from Cortiment^®^, Entocort^®^, and coated 3D printed caplets. (B1) Rendered images of shell-core (top), shell-core delayed-onset sustained-release tablet printed by HME-FDM process (middle), and SEM images of the tablet’s surface (down). Reproduced from [27], with permission from Springer Nature. (B2) Dissolution test of 3DP tablet in USP II pH change solutions. (C1) Experiments on a pressure-controlled drug delivery system for 3D printed capsules printed by HME-FDM process. Reprinted from [28], Copyright (2019), with permission from Elsevier. (C2) Cumulative release of APIs from 3DP capsules. (D1) Photographs of blank NPs, compressed tablets containing OP-NP, and 3DP tablets containing OP-NPs. Reprinted from [29], Copyright (2022), with permission from Elsevier. (D2) Cumulative release of APIs from 3D printed capsules.

Goyanes *et al* used a combined process of HME-FDM and fluidized bed to prepare DR-DFs of budesonide (figure 8(A)). Firstly, budesonide was mixed into polyvinyl alcohol (PVA) filament using the HME process. These filaments were then used to print capsules containing 9 mg budesonide in the FDM 3DP process. Finally, using a fluid-bed coater, Eudragit^®^ L100 was used to coat the 3DP tablets. The initial release of the model drug from the 3DP tablets was triggered in the mimic environments of the middle small intestine and subsequently sustained release (SR) in those of the distal intestine as well as a colon in an in vitro release assay, data of which were compared with those of two commercially available budesonide products, Cortiment^®^ and Entocort^®^ [26]. The authors demonstrated for the first time the possibility of integrating FDM 3DP with existing pharmaceutical technologies, such as HME and thin-film coatings, to prepare oral DR-DFs.

Okwuosa *et al* used a dual combination of HME and FDM 3DP to print an enteric-coated tablet with a shell-core structure for delayed release (figure 8(B)). After the prescription screening, the mixture of Eudragit L100–55, TEC, and talc (w/w: 50%, 16.67%, and 33.33 %, respectively) was extruded based on the HME process to make shell filaments for FDM printing. Eudragit L100–55 is a methacrylate-ethyl acrylate copolymer (50: 50) generally chosen to prepare enteric solid DFs. Its uniqueness of structure makes it dissolve in a pH-response way, immiscible at the stomach’s physiological pH while soluble in pH 5.5 or above media. The mixture proportion, especially for TEC, used in the formulation can also not be ignored, significantly reducing the Tg of Eudragit L100–55 and facilitating the continuous and smooth printing of filaments through FDM 3DP. Theophylline as a model drug, PVP as a polymeric matrix, TEC as a plasticizer, talcum powder as a filler, and core filaments for FDM 3DP were also obtained based on the HME process. Double FDM 3DP technology could control the drug release of shell-core structure tablets by regulating the shell thickness. After the dissolution test at the varied pH conditions, it was confirmed that a shell thickness of 0.52 mm or higher could protect the drug-containing core in acidic media. To prove the system’s applicability to different APIs, budesonide or diclofenac sodium were also selected as model drugs to be mixed into PVP filaments to prepare the tablet’s core. Then, similar results were achieved [27].

In the fasted state, the stomach can not digest non-digestible solids. Gastric contractions (phase III) lead to intragastric pressure up to approximately 130–200 mbar, pushing solid medicine out of the stomach. The pressure can rise to as much as 500 mbar during gastric emptying [175]. Krause *et al* utilized this unique physiological property of the GI tract to design pressure-controlled capsules based on combining HME-FDM 3DP (figure 8(C)). The relevance tests of dissolution pressure showed that no acetaminophen (model drug) was released when 200 mbar of pressure was applied to the measured DFs. In contrast, the drug in the capsules began to release when the pressure reached 250 mbar. 85% of the drug in the capsules was released within 8 min after crushing [28]. This study introduced a new concept for oral drug delivery: pressure-governed DFs produced by 3DP. The most important task of this experiment is to optimize the printing parameters to make capsules that meet the physiological characteristics of the GI tract.

The implementation of site-specific drug delivery in the GI tract or prevention of drug degradation in harsh gastric environments is of utmost importance for DR-DFs. Recently, Mirdamadian *et al* combined nanotechnology, HME, and FDM 3DP to print a colon-targeted oxaliplatin sheet (OP, figure 8(D)). Printable filaments containing Eudragit L100–55 mixed with the loaded OP-alginate nanoparticles (OP-NPs) were achieved by HME. Cylindrical tablets with these filaments were printed using FDM technology, which has good drug uniformity in drug loading efficiency and selectively releases OP in a colonic environment. Compared with conventional OP-NP compression tablets, Injections containing OP or oral solutions of OP, the 3DP tablets containing OP-NPs showed more substantial anti-tumor effects and better safety in CT-26 tumor-bearing mice [29]. This study showed that colon-targeted OP tablets prepared by combining nanotechnology and FDM 3DP could be used as a reasonable method to treat colon cancer due to better antitumor activity, improved tumor targeting and safety.

### 3.3. Sustained release of dosage forms

SR-DFs refer to formulations that can continuously release drugs for an extended period after medication to achieve long-lasting effects [176]. SR-DFs offer some therapeutic advantages, such as (1) releasing the drug at a relatively constant rate to keep the stable drug concentration within the therapeutic range, (2) avoiding the potential risk and inefficiencies caused by ‘peak and valley’ phenomenon of conventional formulations, and (3) reducing the exposed amount of drug and its side effects [177]. Generally, SR-DFs have three primary forms: first, DF using a biocompatible polymer as the matrix (monolithic matrix devices), second DF using diffusing rate-controlled membranes (film coating reservoir devices), and third DF depending on osmotic pressure (osmotically controlled devices). The key to these DFs is to select appropriate polymers. The ideal polymer drug carriers should be non-toxic, biocompatible and biodegradable, have good drug loading capacity, penetrate the desired site of action, and release the APIs in a controlled way [178, 179].

**Figure 9.**
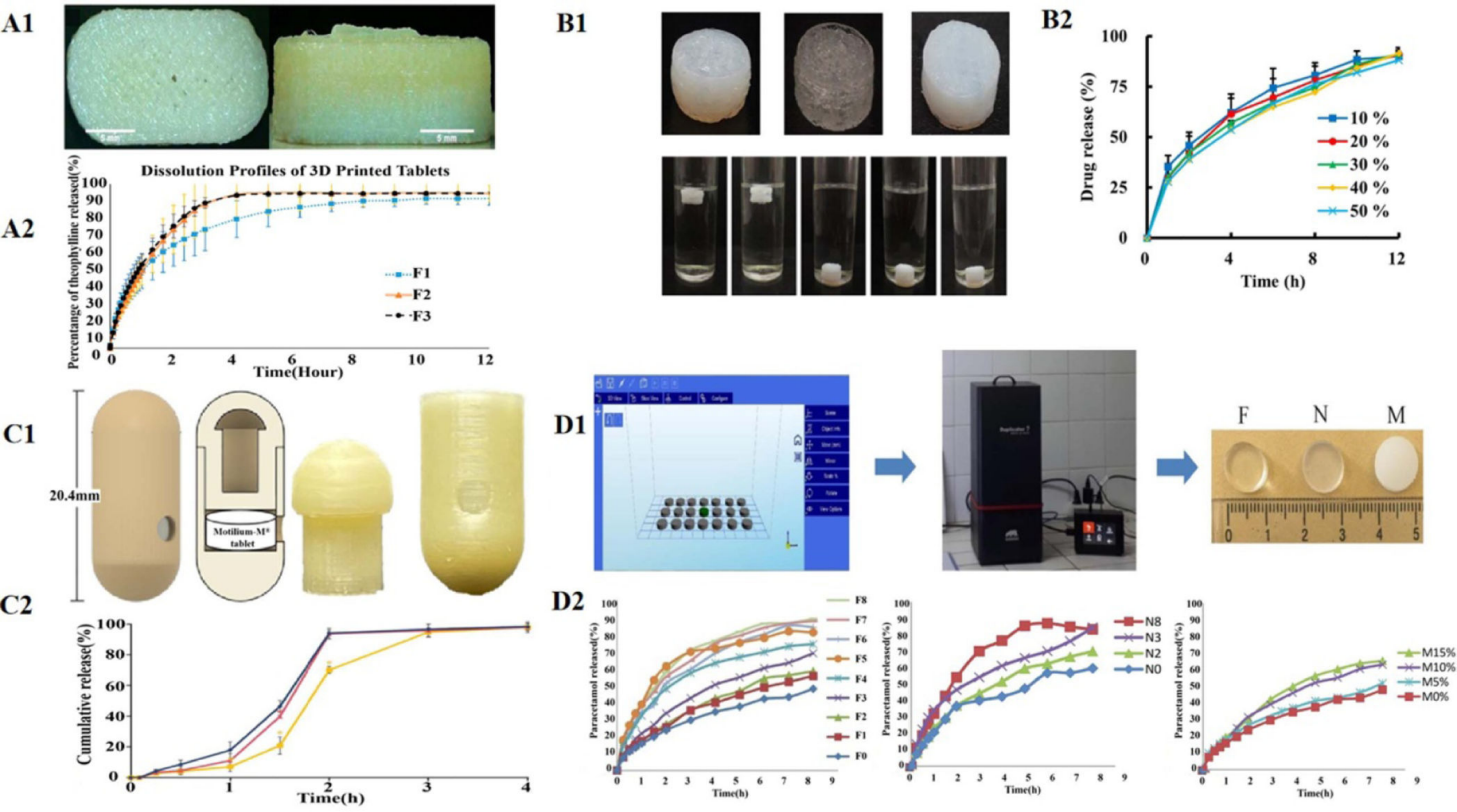
Examples of sustained release dosage form of 3DP. (A1) 3DP tablet printed by HME-FDM process. Reproduced from [34]. CC BY 4.0. (A2) Dissolution profiles of the 3DP tablets. (B1) Actual rendering of 3DP gastric floating tablets prepared by FDM 3DP process. Reprinted from [183], Copyright (2021), with permission from Elsevier. (B2) *In vitro* release curve of tablets. (C1) Illustrative designs and entity of capsule-shaped 3DP devices prepared by the FDM 3DP process. Reproduced from [40], with permission from Springer Nature. (C2) *In vitro* drug release in artificial gastric fluid. (D1) Flow chart for preparation of printed tablets. Reprinted from [42], Copyright (2019), with permission from Elsevier. (D2) *In vitro* release curves of different formulations of tablets prepared by photopolymeric digital light processing (DLP) methods.

3DP has been extensively applied in the studies of the controlled release of DFs. Tablets prepared by SLS 3DP usually dissolve and release APIs within minutes due to their inherent porous and loose structures. Giri’s group was the first team to show that Kollidon^®^ SRK could be utilized as an appropriate polymer substrate to explore the slow-release formulations using SLS 3DP. These DFs could continuously release about 90% of acetaminophen within 12 h. No burst-release phenomena occurred compared to free drugs or physical mixtures containing APIs [180]. The FDM 3DP uses many polymers with high Tg, which makes it ideal for manufacturing DFs with sustained release (SR) properties [30–33, 36, 181, 182]. Tan *et al* Developed the manufacturing platform to print a cost-effective patient-tailored DDS using the HME-FDM 3DP method [34]. Taking theophylline as a model drug, HPC, Eudragit^®^(RL PO), and PEG as excipient polymers, an SR capsule (PrintCap) was printed using the HME-FDM process (figure 9(A)). The data from the in vitro drug release confirmed that the SR time was over 10 h. Approximately 80% of the API was released from the printed capsules, which was tuned by the varied ratios of polymers used but not by the printing parameters.

By changing the composition or geometry of tablets, filling percentage, outer shell thickness, and capabilities such as floating and controlled release to adjust the density and hardness of tablets, FDM 3DP is used to prepare novel oral DFs, such as intragastric floating SR-DFs (figure 9(B)) [35–39, 184, 185]. Most of these intragastric floating DFs were ready by the HME-FDM method. However, only heat-resistant drugs can be mixed into filaments by the HME process. Therefore, this method can not prepare thermally unstable drugs [186]. To solve this problem, Charoenying *et al* prepared a capsule of 3DP device (CPD) for intragastric floating drug delivery by FDM using PVA as a printing filament (figure 9(C)). The CPD was mainly composed of two parts: cap and body. To realize the flotation of DFs in the stomach, their density could generally be less than that of the stomach contents (1.004 mg mm^−3^) [183]. Therefore, to realize the aim of floating in the stomach, the hat was designed as a structure with air space. On the other hand, the body structure was mainly composed of three parts: (1) solid heavy bottom to achieve the upright floating of CPD; (2) drug-releasing hole with a diameter of 2.5 mm; (3) the jointing part of the cap and body was left with space enough for filling drug powder. The RSM method was subsequently used to optimize the size of the CPD to provide a gastric retention drug delivery device with optimal suspension time [40]. The photopolymerization 3DP technology forms highly cross-linked polymers as a highly dense, non-porous and compact matrix, ideally suitable for preparing sustained-release DFs [41, 187]. When in contact with a medium, these polymer matrices expand, and the API diffuses through the augmented matrix in a slow-release manner [188].

SLA process is mainly applied in the development of hydrogels. The properties of the matrix are closely related to the molecular weight and concentration of the selected polymer, which affects the density of the hydrogels formed [189, 190]. The *in vitro* studies of hydrogel drug release showed that the release speed depended on the water content in the gel. The higher the water content in the gels, the faster the drug release rate [191]. Taken diphenyl (2,4, 6-trimethyl benzoyl) phosphine oxide as a photoinitiator, PEG diacrylate (PEGDA) as a photopolymer, and paracetamol (acetaminophen) as a model drug, Krkobić *et al* printed SR tablets using photopolymeric DLP 3DP technology (figure 9(D)). The release rate was increased by adding hydrophilic polymers such as PEG 400, sodium chloride (NaCl), and mannitol. Most tablets exhibited SR features over 8 h [42].

### 3.4. Pulsatile release of dosage forms

PR-DFs refer to the formulations that can release the required amount of drugs according to the characteristics of pathophysiological time rhythms. APIs can be released to relieve or control the symptoms at a specific time when certain diseases are prone to attacks [192]. The peak symptoms of many chronic diseases, such as rheumatoid arthritis, bronchial asthma, hypercholesterolemia, and cardiovascular disease, follow a 24 h cycle known as a circadian rhythm. The optimal treatments for these diseases require DDS, in which the Cmax of the drug is precisely reached at the peak of the disease based on a pulsatile delivery technology. These DFs not only synchronize the circadian rhythm with the symptoms of this disease but also overcome the problems of drug tolerance and excessive dosage existing in traditional SR or controlled-release DFs. The PR-DFs have the advantages of programmable SR drug delivery, multiple drug delivery capability, reduced drug side effects, and improved patient compliance [193]. Due to its ability to produce complex geometry, 3DP formulation may be an alternative to the traditional pulse-release counterpart. Therefore, 3DP may become a more straightforward and practical approach to realizing this goal [194, 195].

**Figure 10.**
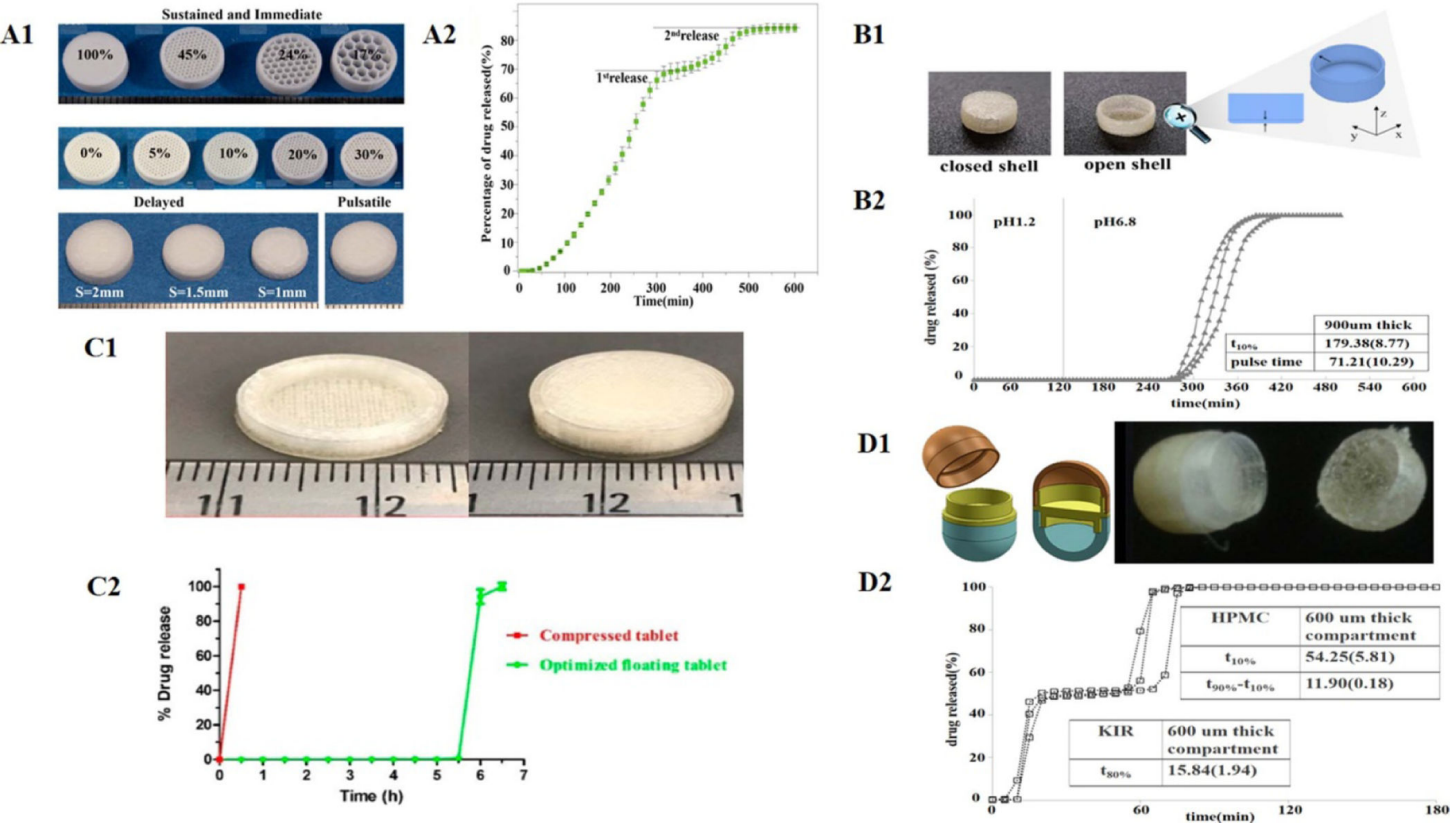
Examples of pulsatile drug release dosage form of 3DP. (A1) Images of tablets with different release behaviors prepared with a novel dual-nozzle solvent-free hot-melt inkjet 3DP system. Reproduced from [43]. CC BY 4.0. (A2) *In vitro* dissolution test of pulsatile release tablets. (B1) Images of pulsatile release—Chronotopic^™^ system printed with a dual-nozzle FDM 3D printer. Reproduced from [44]. CC BY 4.0. (B2) *In vitro* release curve of APIs in the different solutions (pH, 1.2 and 6.8). (C1) Images of novel core-shell gastro retentive floating pulsatile DDS printed by HME-FDM 3D printer. Reproduced from [45]. CC BY 4.0. (C2) *In vitro* release profiles of the floating tablets. (D1) Schematic and solid drawings of 3D printed multi-compartment capsules printed by HME-FDM 3D printer. Reprinted from [46], Copyright (2017), with permission from Elsevier. (D2) *In vitro* release profiles of capsular devices.

Lion *et al* developed a novel dual-nozzle solvent-free hot-melt inkjet 3DP system to design and print multiple DFs, meeting the requirements of customized drug release (figure 10(A)) [43]. Using Compritol HD5 ATO (styrene polyoxy-8 triglyceride) as a matrix and fenofibrate as a model drug, the authors successfully printed pulse-release tablets by introducing two concentric regions containing the drug into a drug-free matrix to form core-shell structures. In addition, a different ratio of surface area to volume (SA/V) was given to the tablets by adjusting the filling rate, which influenced the release rate of the drugs. The slow-release tablets (100% filling) and fast-release tablets (45% filling) were successfully prepared. The authors also designed the delayed-release tablets with an external drug-free (barrier) layer to release the drug rapidly after the initial delay, and the release could last 12 h. Melocchi *et al* chose a duplex-nozzle FDM 3D printer to print a pulse-release Chronotopic^™^ system [44]. The system has a double core-shell structure consisting of a drug-containing layer (inner core) and a polymer barrier layer (outer shell, figure 10(B)). The drug-free or a drug-containing core part of formulation (e.g. powder, pellet, and gel) was manufactured by FDM, while the shell consists of an expansible/soluble hydrophilic polymer that can form a slowly dissolving/eroding gel layer when interacting with the medium. Therefore, the shell thickness could regulate the delay time (T 10%) and release time (pulse time). In addition, the authors added an external anti-gastric acid coating to achieve a three-layer colon-targeting DFs. Asthma symptoms get worse in the morning because of the circadian rhythm. Therefore, Dumpa *et al* utilized the HME-FDM 3DP technology to produce novel intragastric floating theophylline-loading pulsatile tablets (figure 10(C)). The tablet was also a shell-core structure, and the delay time was regulated by shell wall thickness, filling density as well as the ratios of ethyl cellulose (EC) (*P <* 0.05). Therefore, the delay time can vary from 30 min to 6 h as needed. The data from the in vitro dissolution tests have confirmed that the gastric floating tablet with parameters including a 0.2 cm shell, 0.16 cm wall thickness, 0.5% EC and 100% infill was considered the best pulse-release prescription, effective in treating asthma [45].

Maroni *et al* chose the HME-FDM 3DP method to print multi-compartment double-pulse release capsules (figure 10(D)). The capsule was divided into three chambers: a rapidly soluble chamber, a gastric acid-resistant chamber, and an expansible/erodible chamber, which enabled the rapid release of tablets in the stomach or pulse release of those in the intestine tract, respectively. Thus, the different chambers of the capsule were constructed with varying compositions of material and varied wall thicknesses, endowing the DFs with multiple release kinetics features. In addition, capsules could be filled with different doses of APIs and their formulations, which would satisfy the needs of other patients, significantly easing the personalization of drug treatment [46].

### 3.5. Polypill and personalized combination

Polypill is the mixture of several APIs into one preparation, each component having its pharmacological effect. Polypill aims to pursue the effectiveness of combination therapy, simplifying treatment plans and reducing production, transportation, and storage costs. Last but not least, the formulation can improve medication compliance [196]. However, the production process of traditional compounds is highly complex, and more importantly, the conventional ‘one-fit-all’ compound ignores personalized needs [197]. Due to the differences in bioavailability and medication demand among children, adults, and the elderly, patients may need personalized formulations with different formulas and dosages [198]. As an emerging production method, 3DP has the advantages of fewer processing steps, lower cost, and more flexible design in the on-demand production of personalized medicine, which is very suitable for producing multiple API preparations [199–202].

**Figure 11.**
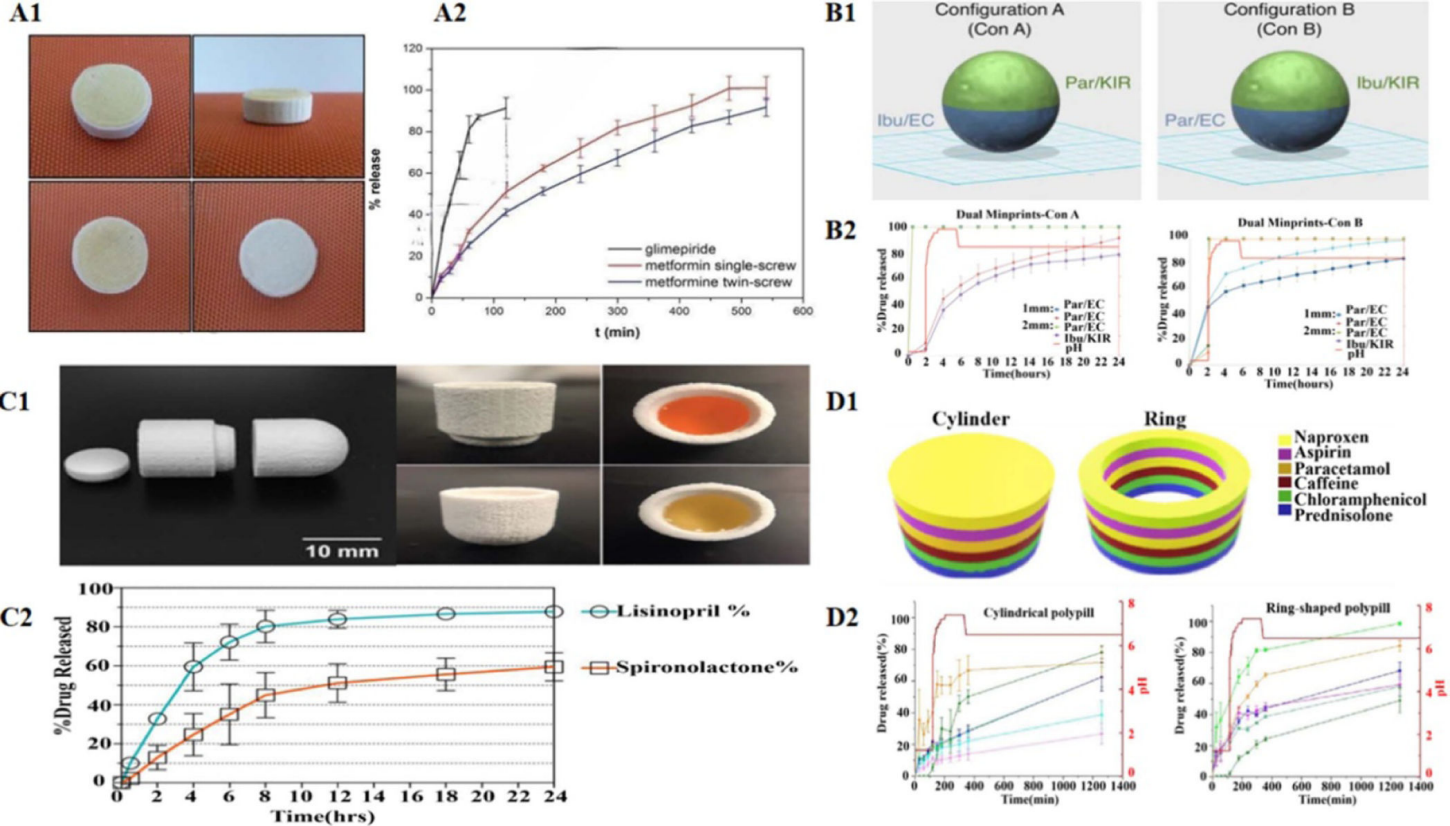
Examples of personalized combinations and polypills printed by the combination of HME-FDM (A), SLS (B), adhesive piezoelectric jet printing process (C), and SLA (D). (A1) A 3D printed tablet. Reprinted from [47], Copyright (2018), with permission from Elsevier. (B1) Schematic representation of the distribution of configuration (A) and (B) compositions within the dual miniprintlets. Reproduced from [51]. CC BY 4.0. (C1) Multi-compartment preformed tablet. Reproduced from [53]. CC BY 4.0. (D1) Schematic representation of a multi-layer compound 3D printed tablet containing six drugs. Reproduced from [52]. CC BY 4.0. (A2, B2, C2, and D2) *In vitro* release curves of APIs from these dosage forms.

Because specific APIs in the compound formulations have different release kinetics and absorption positions, multiple different release modes for APIs in the formulations are very beneficial to meet the requirements of clinical therapy. These factors should not be ignored when developing compound formulations using 3D printing technology. For example, among the drugs used to treat type 2 diabetes, metformin is mainly absorbed in the small intestine, while glimepiride is absorbed in the stomach. To ensure the most optimal treatment outcomes, Gioumouxouzis *et al* used HME-FDM 3DP to develop a two-layer anti-diabetes formulation with different release profiles of two model drugs: glimepiride (IR) and metformin (SR), which made it possible to administer both regimens simultaneously and once daily (figure 11(A)) [47]. Certain diseases, such as Parkinson’s disease, can only be treated symptomatically, and the dosage must be very accurate for patients. The combination of levodopa (LD) and dopamine decarboxylase inhibitors is a first choice for Parkinson’s disease, supplemented by dopamine agonists simultaneously. Based on the specific requirements of these treatments, Windolf *et al* used FDM 3DP to print stomach floating polypill to treat Parkinson’s disease [48]. LD, benserazide (BZ), and pramipexole (PDM) were chosen as model drugs in their studies. The authors used HME to prepare two drug-loaded filaments: PDM and PVA for the IR part and BZ/LD (1:4) in ethylene-vinyl acetate copolymer matrix (SR). The FDM was used to print this multiple-compound-containing preparation to achieve personalized treatment of on-demand drug delivery by regulating the complexity of shapes and changing different ratios of two filaments. Meanwhile, a miniature gastric-floating compound preparation was printed to satisfy the requirements of patients with dysphagia, which released three-quarters of BZ/LD within 12.5 h.

FDM is the most extensively applied and cost-effective printing technology. However, the low drug loading capacity and the instability of heat-sensitive drugs severely limit its application in the pharmaceutical industry. Researchers have been exploring possibly combining other technologies with FDM to overcome these limitations. For example, Keikhosravi *et al* first used HME-FDM 3DP and melt casting techniques (MCTs) to prepare aspirin/simvastatin DR-DFs to treat cardiovascular diseases [49]. The low temperature and solvent-free conditions of the MCT process ensured the integrity of the compound preparation. For compound formulations containing aspirin and statins, incompatibility between two APIs is critical in the preparation process. Therefore, the physical separation of two APIs in compound formulations is beneficial. The author used FDM-3DP to prepare the tablet with a dual chamber structure, ensuring absolute separation to keep the stability of incompatible drugs. The authors showed that combining HME-FDM and MCT significantly improved the drug-loading capacity of 3DP compound preparations. For patients suffering from chronic cardiovascular diseases such as hypertension and hyperlipidemia, most of them usually need to take two or more medications to achieve optimal treatment outcomes [203]. The complex therapeutic regime always causes many problems, including psychological distress, depressive symptoms, and poor patient compliance [204–206].

Compound formulations may be a more simplified and effective strategy. However, the rigid requirements for fixing formulas containing multiple drugs in traditional compound formulations may only meet a limited number of eligible patients. However, polypills prepared by 3DP, due to their design flexibility, can be developed according to the needs of patients by manufacturing a dynamic dose combiner [207]. Pereira *et al* used FDM aligned with hot-filling syringes to create a multi-compartment capsule simultaneously loaded with four model drugs for treating cardiovascular diseases [50]. The capsule was designed as an oval hollow geometric structure containing four compartments, and different drugs were loaded into each isolated compartment to avoid potential drug–drug interactions. The compartments were set up in two predesignated structures (concentric or parallel), and customized drug release was achieved by controlling the wall thickness of the shell in the concentric structure and the hole size in the parallel structure.

Awad *et al* are the first group to use SLS 3DP to produce multi-particle oral DFs containing paracetamol (Miniprintlets) (figure 11(B)) [51]. They also developed Miniprintlets containing paracetamol and ibuprofen. The redesign of the compound Miniprintlets was achieved by changing the polymer prescription to meet the needs of customized drug release, where one drug was released from the Kollicoat matrix for IR requirements, and another was released continuously from EC for SR needs. Miniprintlets with different doses but the same proportion could meet the varied needs of patients of different ages, especially young and elderly. Acosta-Velez *et al* designed and manufactured combined oral DFs with two chambers to treat hypertension through the adhesive piezoelectric inkjet 3DP (figure 11(C)) [53]. The formulation used a photocurable hydrophobic bio-ink of PEG and a photocurable hydrophilic bio-ink of hyaluronic acid mixed with lisinopril and spironolactone, respectively. The formulations with varied bio-inks were then polymerized under visible light. The in vitro dissolution studies showed that lisinopril and spironolactone could achieve double SR profile within 24 h. Most binder jetting printers have been developed for mass production, which is unsuitable for small-dose personalized drug delivery, let alone in the development of compound formulations. However, Lu *et al* used an on-demand inkjet printing process to develop solid and concentric cylindrical tablets containing three model anti-viral drugs (favipiravir, hydroxychloroquine-HCS sulfate, and ritonavir). The in vitro drug release studies showed that the released API in the external layer was more than 90%, that in the intermediate layer was more than 70%, and that in the inner part was only 40%. The data indicates that the external and intermediate layers are appropriate for immediate release, whereas the inner part can be used for delayed release. In addition, the advanced surface analysis using micro CT imaging, Artificial Intelligence and Deep Learning model validation exhibited that the drug was evenly distributed in the printed tablets, even at extremely low doses [208]. The slow application progress of SLA 3DP in compound formulations is due to the limitations of commercial printer hardware and software in spatially separated layers. However, Robles-Martinez *et al* prepared an oral polypill using the SLA 3DP process (figure 11(D)). The DFs were loaded with six drugs: caffeine, paracetamol, prednisolone, chloramphenicol, naproxen, and aspirin [52]. The authors modified a commercial SLA 3D printer and optimized the software to stop printing during the printing process. Then, they removed the resin pallet and interchanged it with a different drug-containing resin filament. Multilayer compound preparations were successfully prepared with different geometric shapes (cylindrical and annular) and chemical compositions (without or with soluble filler). Loading six model drugs into different materials could achieve different drug release profiles in the dissolution test. This demonstrated that SLA printing was a feasible groundbreaking platform for polypill production for the first time, which promoted the development of 3DP in personalized polypill. 3DP may change the future of compound formulations; drugs used by the patients can be printed into one pill to reduce the frequency of drug intake, and each API has its customized release profile. But before that, an excellent clinical control study is needed to ensure clinical therapy’s safety, avoiding drug–drug and drug-polymer interactions [209–211].

### 3.6. Microneedle

MNs are arrays of micron-sized needles that penetrate the stratum corneum but do not touch nerve endings below the epidermis, delivering therapeutic drugs across the skin without causing pain [212]. As a novel transdermal drug delivery system (TDDS), MN provides a minimally invasive administration method to deliver various APIs safely, painlessly, and easily manipulated due to the formation of microscale channels in the skin [213, 214]. There are five main MN delivery strategies: solid, coated, dissolved, hollow, and hydrogel formation [215]. Most traditional MN-manufacturing methods have to face the problems of complex multi-step manufacturing processes, labor, time consumption, and expensive equipment, which have greatly limited the industrialization and personalization of multifunctional MN [216]. The emergence of 3DP technology ideally overcomes the limitations of the above manufacturing methods, enabling rapid and continuous one-step forming and personalized customization of MN [217].

**Figure 12.**
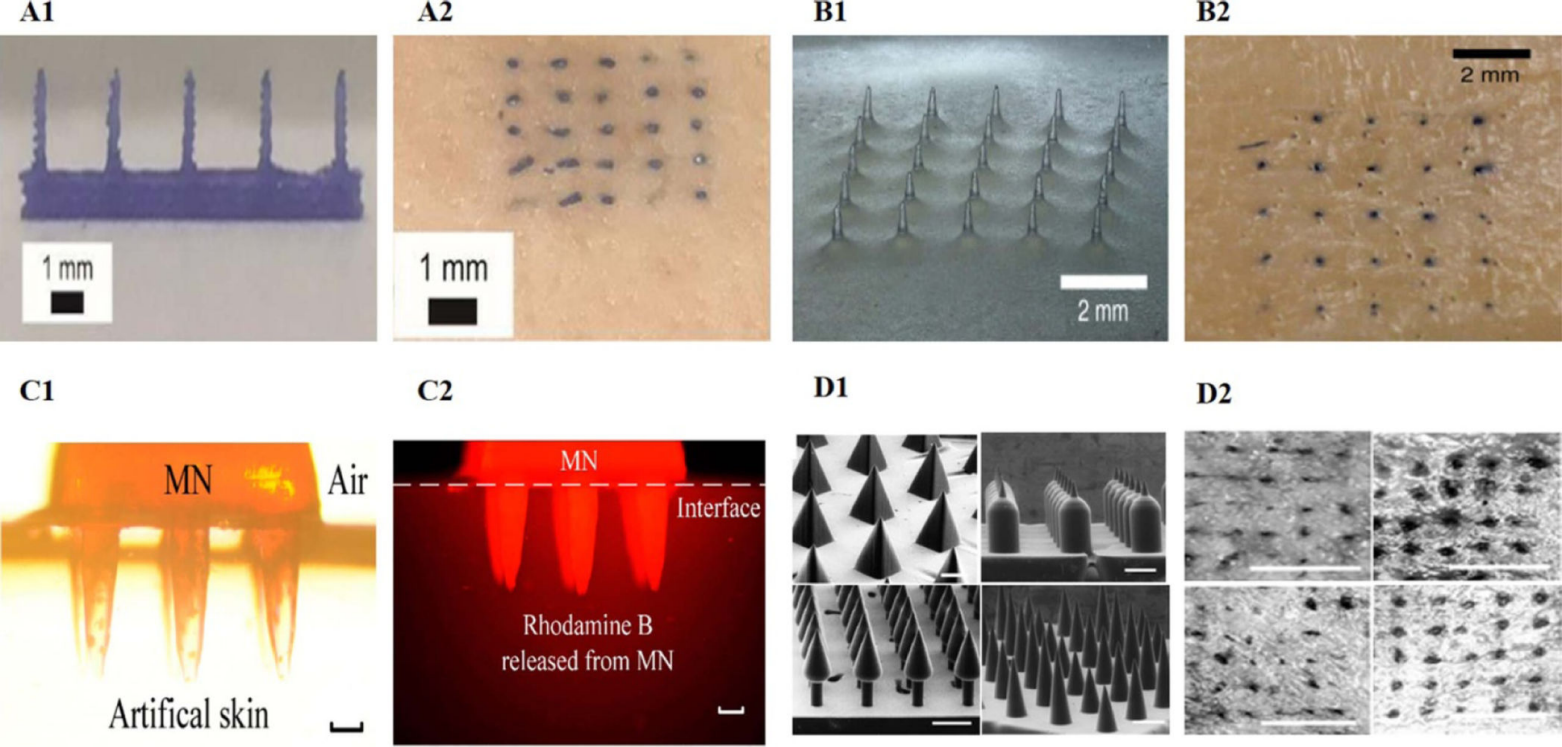
Examples of 3DP microneedles. (A1) Image of the FDM microneedle. Reproduced from [56] with permission from the Royal Society of Chemistry. (B1) Image of the SLA microneedles. Reproduced from [59]. CC BY 4.0. (C1) DLP microneedles were implanted into artificial skin. (C2) Fluorescence images of MNs containing rhodamine B stuck into artificial skin without this dye. Reproduced from [61]. CC BY 4.0. (D1) CLIP microneedles with different shapes. Reproduced from [62]. CC BY 4.0. (A2), (B2), and (D2) Skin insertion studies of microneedles.

FDM and photopolymerization in 3DP technology have recently been widely reported to manufacture high-quality MN [218–221]. The MN produced by FDM can control the release of multiple drugs through a wireless intelligent control system, achieving an ideal release rate and drug distribution. Derakhshandeh *et al* used FDM to create transdermal MN for Chronic Wounds [54]. The authors loaded vascular endothelial growth factor (VEGF) into MN to deliver the protein to the deep wound bed controlled by a wireless-controlled smart bandage. The data showed that the method of drug delivery and its spatial distribution within the affected tissue is as vital as selecting appropriate therapeutic agents. Tang *et al* selected PLA as a polymeric matrix to produce a TDDS with milliprojections by FDM 3DP, a modified preparation of MN [55]. By evaluating the milliprojection of surface finish and dimensional accuracy, the authors held that FDM printing parameters, including printing temperature, layer thickness, extrusion width, filling width, and nozzle hole diameter, significantly affected the final quality of the milliprojection. However, compared with other 3DP methods, the printing resolution of FDM is the lowest. The object produced by FDM has inherent anisotropic, uneven strength, poor dimensional accuracy, and rough surface. To overcome these problems, Luzuriaga *et al* combined FDM 3DP with a chemical etching post-processing method to print high-precision biodegradable MN (figure 12(A)) [56]. The data showed a new chemical etching method could fabricate MN with tip sizes ranging from 1 to 55 *μ*m. Various MN shapes could be rapidly designed and manufactured using an FDM 3D printer, the density and length of which can be customized.

Additionally, Camović *et al* produced biodegradable PLA MN by the same process [57]. The authors further investigated the possibility of coating the printed MN surface with API. The results showed that the ideal size and shape of MN could be customized by this method. The dip coating method is a top-notch drug-loading process for 3DP MN due to its simplicity and capability to evenly coat API onto the MN surface. Due to weak binding between PLA layers during FDM 3DP, PLA MNs may be separated before chemical etching to the desired size. Increasing the thermal parameters in the FDM 3DP process (i.e. the temperature of the printing stage and nozzle) is an excellent choice to improve the interface binding strength between MN layers, which can effectively prevent PLA MN shedding and base collapse during the corrosion process. In addition, increasing the concentration of the etching agent and processing temperature can improve the efficiency of chemical etching [222].

The high resolution provided by SLA 3DP guarantees the fabrication of hollow MN for transdermal drug delivery, which has high fidelity, reproducibility, ease of implementation, short production time, and relatively low processing costs [223]. Yadav *et al* used SLA 3DP technology to design and manufacture hollow MN connected to reservoir voids [58]. The MN structure consisted of a solid tip with the pinhole forming an angle of 90° with the direction of the tip (side opening). The hollow base of the MN was used as a storage chamber to load the drug solution. Both in vitro, permeation experiments on pig skin and in vivo experiments in SD rats showed that rifampicin-loaded SLA MN had good permeability and bioavailability. Before universal MN with miniature scale could be precisely manufactured, the most significant challenge was fabricating controllable molds, which was essential but often expensive and time-consuming for subsequent MN preparation [224]. Krieger *et al* used a cheap desktop SLA 3D printer to fabricate molds through a new two-step ‘print & fill’ process (figure 12(B)) [59]. The method was simple to operate, which required neither expensive nor complex equipment nor expert knowledge in micromachining. Still, the resulting higher aspect ratio of MN molds was pleasing. The quality of MN can be effectively improved by optimizing the machining and manufacturing process parameters, guaranteeing sufficient accuracy.

Choo *et al* manufactured high-resolution, high-dimensional MN molds after optimizing SLA 3DP parameters [60]. Adjusting the aspect ratio, needle height, printing angle, and MN spacing of SLA, especially when the printing angle of the *X* and *Y* axes was 60°, the sharpest tip MN could be printed in a single overlapping layer area. MN molds prepared under optimal 3DP conditions could fabricate soluble MN with the mechanical strength of penetrating skin by solvent casting. In addition, they could produce side-notch arrow MN with high-dimensional structures by adjusting the 3DP angle. DLP 3DP is superior to other 3DP technologies in rapidly producing MN with smooth surfaces and high resolution [225, 226]. Yao *et al* prepared a hydrogel MN using a DLP 3D printer (figure 12(C)) [61]. The sharp protrusion and pores of the hydrogel MN enabled them to perform multiple goals, including drug delivery and substance detection, in a minimally invasive manner. By changing the cure time of each layer, satisfactory mechanical properties such as enough stiffness and high precision could be obtained. The authors provided a low-cost and quick-making method for MN. They also verified MN’s mechanical properties, drug administration, and drug detection capability, exhibiting great potential for clinical application.

The unique design of CLIP 3DP avoids using scaffolds during the printing process. It provides faster printing speeds without compromising isotropic mechanical properties, making it suitable for preparing MN with different surfaces and shapes [227, 228]. Johnson *et al* used CLIP 3DP to produce square conical MN consisting of poly(acrylic acid), trimethylpropane triacrylate, and photopolymeric derivatives of PEG and polycaprolactone (figure 12(D)) [62]. The data showed that CLIP 3DP could rapidly generate MN with various aspect ratios, shapes, sizes, spacing, and compositions. These MNs could penetrate the mouse skin and release the encapsulated rhodamine of the API substitute. Caudill *et al* designed and fabricated a multilayer MN for vaccination using CLIP 3DP technology [63]. The authors showed that this MN enhanced the retention of vaccine components in the skin and activated immune cells to trigger more effective humoral and cellular immune responses compared to the traditional vaccination route.

## 4. The benefits and challenges of 3DP in drug delivery

### 4.1. Benefits

#### 4.1.1. Personalization

Pharmacogenetics and pharmacogenomics have become essential pillars of personalized medicine. It will provide feedback on the response of drugs to individual genotypes and achieve personalized precision treatment by tailoring drugs based on the respective genotypes. Certainly, dosage should also be adjusted according to clinical response [229]. Personalized dosing may be the best regimen based on a patient’s body weight, pharmacokinetic parameters, pharmacogenetic characteristics, and other factors, including sex, age, and race. Oral DFs are generally administered using a simple dosing device, such as a dosing spoon or tablet-splitting device. These devices often have problems such as unequal dose distribution, drug failure, and resource waste [230]. 3DP can create personalized DFs with flexible dosages to avoid the issues above and has opened up a new path for developing patient-centered dosage forms, meeting the demand for personalized medication [231, 232]. From infancy to adolescence, the child experiences various physiological states. Children have different requirements for the tolerability and dosage of drugs, so the treatment regime and dosage may need to be continuously adjusted. Additionally, the taste and palatability of drugs are also vital factors in improving children’s compliance [233].

The high flexibility of the dose of 3DP drugs and attractive appearance through simple changes in size, filling density, and digital shape meet the requirements of personalized pediatric formulas, improving treatment compliance and reducing diseases’ impact on children’s emotions. Generally, older adults are more prone to organ changes related to the clearance of chemotherapeutics (such as the kidneys). They are susceptible to certain compounds (such as psychotropic drugs and atropine drugs). Therefore, it is usually necessary to adjust medicines to adapt to ensure the safety and effectiveness of different dosages. Last but not least, elderly patients may be in an overdose treatment state after taking large amounts of medication (sometimes even more than ten medications) every day [197].

Medication error rates have been reported as high as 35% among patients who take more than four medications [234]. Combined use of multiple drugs daily among older adults may lead to adverse outcomes such as poor compliance, adverse reactions, drug–drug interactions, and the increased risk of hospitalization [235]. Another application of personalized dosing is printing multidrug combinations, called ‘polypills.’ The dosage and composition in the DFs can be modified by 3DP, which is very suitable for the personalized needs of elderly patients. The contour of a 3D-printed DF is not determined by the shape and size of traditional manufacturing tools (i.e. presses and dies). 3DP can design flexible shapes of DFs. Since drug structure affects drug release, the complex structure and geometry shape of 3DP drugs create new possibilities for API release [236]. A significant factor affecting drug release in vitro is the tablet surface area/Mass (SA/M) ratio. 3DP can change the SA/M ratio differently, resulting in the formulation with different release profiles [237]. This will be very suitable for drugs characterized by narrow treatment windows, in which formulation precisely controls the dose, leading to safe release kinetics. Indeed, the process technology of the 3DP still needs to be further validated. For example, the reproducibility and accuracy of dosage may be affected by printing resolution or mixing uniformity of APIs and excipients.

Traditional manufacturing processes are unsuitable for personalized medicine because they can not fabricate customized DFs with modified release profiles and complex geometry structures. Personalized medicine requires a higher manufacturing process for DFs, which can be digitally manipulated using 3DP technology and dosages can be regulated through software commands. Based on 3DP technology, people can design proper shapes to meet the needs of patients with dysphagia [238]. 3DP technology makes it easier to achieve personalized medicine. Changing a digital design is much easier than modifying a physical device. However, the application of 3DP in personalized medication is still in its infancy. It faces many problems, such as the availability of non-toxic materials, medication safety, regulatory ambiguity, quality control standardization, etc. There is a long way to go before realizing 3DP personalized precision medicine.

#### 4.1.2. Economical and logistics in the field of pharmaceuticals

In the pharmaceutical industry, 3DP’s vision includes transitioning drug production from traditional centralized (pharmaceutical factory) to decentralized (such as in hospital clinics, local pharmacies, and even at patient homes) [239]. Therefore, 3DP may help break away traditional mass manufacturing and shift to mass customization or on-demand production [240]. Decentralized drug production will bring many benefits: firstly, this model can reduce the waste of time and economic costs in the transportation and storage of drugs between different regions. A vast database may be formed to store the required manufacturing files. Patients can download the files from cloud data, or doctors can send them to patients worldwide through the network. And finally, patients can print them on demand at nearby medical facilities. This will reduce the demand for transportation, storage, and mass manufacturing processes and reduce the consumption of energy sources. Importantly, it is closer to consumers so that it can respond sensitively and promptly to the needs of patients. The on-site production of 3DP is suitable for customizing formulas according to the conditions of the patient’s body.

The decentralized model in 3DP can also be applied to uneasy-to-delivery areas (disaster areas, extremely undeveloped countries, and even space) and emergencies with limited time or resources (operating rooms or ambulances, etc) [241]. Despite the benefits above, it is foreseeable that shifting centralized drug production to decentralized production (such as in healthcare settings or at patient homes) may raise some regulatory, legal, and ethical issues. Even more challenging is that this will bring new problems, such as the security of data transmission, storage and transportation of 3DP materials, the risk of counterfeit production, and quality control methods adapted to 3DP on-site manufacturing to ensure drug safety. From an economic perspective, the centralized, traditional output may be more cost-effective for the large-scale production of conventional drugs, while the production efficiency of 3DP may be challenging to achieve an equal level to mass production. However, personalized specific requirements of patients, regulation of inner structure, shape, size, and dose of formulation by 3DP can meet patients’ treatment needs and preferences. All rules can promote the subjective initiative of patients during the treatment, thereby improving drug compliance and treatment effectiveness and reducing the waste of medical resources [242].

### 4.2. Challenges

#### 4.2.1. Polymeric materials and printing process

Polymers are the material base for preparing 3DP DFs due to their capability to regulate release rates and provide physical stability for the loaded APIs. The type of the chosen polymer depends on the specific 3DP technologies. There is a severe shortage of medical-grade polymer excipients suitable for 3DP, which is a significant dilemma in controlling the release of APIs from 3DP formulations [243]. The feasibility of converting 3D data into 3D objects for the material-extrusion process is primarily determined by the rheological properties of the printed material. Rheological properties are crucial for design fidelity, and appropriate rheological properties may be highlighted to regulate the uniform distribution or even crystalline states of APIs [77]. Although this statement is widely accepted, a lack of knowledge on how to screen 3DP materials and the guidelines for 3DP materials is still a universal and vital concern. Due to the low cost and relatively simple operation of printers, FDM is the most widely studied technology in the pharmaceutical field. However, the main drawback of FDM is the limited number of printable materials. FDM printing needs a drug-loaded thermoplastic polymer wire, which is undoubtedly one of the highly vital steps in the entire process and will increase the production cost of 3DP. High-quality printable wire made from medical-grade polymers as research or industrial raw materials in the market is still rare, while API-loaded cables are unavailable [244, 245]. In addition, the potential risk of drug degradation caused by the high temperature during wire manufacturing and FDM printing processes is another critical issue hindering FDM application in the pharmaceutical industry [246]. The printing material for SSE is a low-melting-point solid or a semi-solid state at room temperature, and the printing process runs at room temperature, thus avoiding the risk mentioned above of API degradation. However, the low resolution of the SSE process and weak mechanical properties of the 3DP object limit its universal applicability in the development of dosage forms. Vat photopolymerization is characterized by high resolution and fast printing speed. Still, its higher production cost and the potential unknown toxicity of printing materials for photopolymerization 3DP seriously hinder the development of technology in the pharmaceutical field. Commonly used polymer resins and PI agents are associated with carcinogenic risk (table 2).

Although the printing materials used in jet printing are generally considered safe, the low mechanical properties of their final products still limit their application to certain DFs that do not require strong mechanical properties, such as IR-DFs. SLS takes a drug-grade powder mixture composed of drugs and thermoplastic polymers as raw materials. Therefore, regarding starting materials, SLS is considered the most similar to traditional tablet-compressing processes compared to other 3DP processes. Like FDM, the heat generated from high-energy lasers during SLS printing may lead to drug degradation and other chemical changes in the powder. Compared with FDM, SSE, VAT photopolymerization, Jet printing, and SLS still need further processing steps such as solidification and drying, prolonging production time, increasing production costs, and adversely affecting APIs’ performance [76].

Material reuse may be a cost-reducing method, but it should be ensured that the reused materials have no adverse effects on 3D printing drugs. Material removal is also a hassle. To make flexible formulation shapes, many internal voids are formed during printing, making it hard to remove the material. Additionally, eliminating support materials from the 3DP structure may result in rough or imperfect printing surfaces. The appearance of DFs is unsightly and may lead to poor patient compliance [124, 250].

The intrinsic shortages of 3DP technology are another challenge for pharmaceutical applications. In a standard 3DP workflow, the CAD design should be converted into a digital file by slicing software, which is next recognized by the printer to start printing. The entire process must ensure that the printer can follow the required manufacturing path accurately and repeatably. Lastly, maintaining data integrity during all file transformations is a top priority [251]. In addition, to ensure the printed 3D objects have the best features, optimizing processing parameters to meet the requirements of the expected applications and printing materials is necessary. Therefore, understanding the impacts of each processing parameter on the printing process and finding the optimal printing parameters is also very important [76].

#### 4.2.2. Regulatory and guidances

The most pressing obstacles facing 3DP in the pharmaceutical industry are the lack of clear regulatory guidance and standard regulations regarding the design, manufacturing process and quality control (QC) of 3DP drugs. In December 2017, the FDA issued a guidance on Technical Considerations for Additive Manufacturing of Medical Devices, which lists the main aspects of quality control, process validation, hardware and software requirements, and equipment testing. Because of the discrepancies among the different 3DP technologies, the FDA document stresses that providing a standard set of guidelines for all 3DP methods is impossible. Therefore, each 3DP technology requires specific regulations [252]. But there are still no rules for 3DP drugs so far, which are supposed to be more urgent than those for medical devices. This will involve many issues concerning liability, safety, intellectual property rights and data protection, and patient medication safety, which must also be ensured. DFs produced by 3DP technology can follow the existing approval pathways. However, at some stage, 3D-printed drugs as new DFs should have unique claims and guidelines [253].

#### 4.2.3. Quality control

Controlling the quality of 3DP products is the biggest challenge. For products produced by traditional manufacturing processes, the quality of each batch is routinely maintained. However, assuming a similar method is used to control the quality variances among batches of 3DP drugs in mass production is problematic. In that case, traditional QC testing is inherently disruptive, laborious, and expensive, which is unsuitable for personalized medicine tailored to individual patients and on-demand manufacturing in small batches [254]. Real-time drug assays and non-destructive QC tests, including process analysis techniques, near-infrared or Raman spectroscopy, or colorimetry, can be performed on 3DP products to verify drug performance, meeting regulatory implementation requirements [255]. Researchers invented tracking measures, namely adding two-dimensional code and data matrices into DFs to realize real-time quality assurance (QA) of drugs to ensure the quality and safety of drugs and promote the application of technology in clinical practice [256, 257]. Unlike QC of batches of traditional drug production, each 3DP drug may have different quality requirements to meet the personalized needs of other patients. Therefore, QA for 3DP medicines is the foundation of finished product quality and safety.

**Figure 13.**
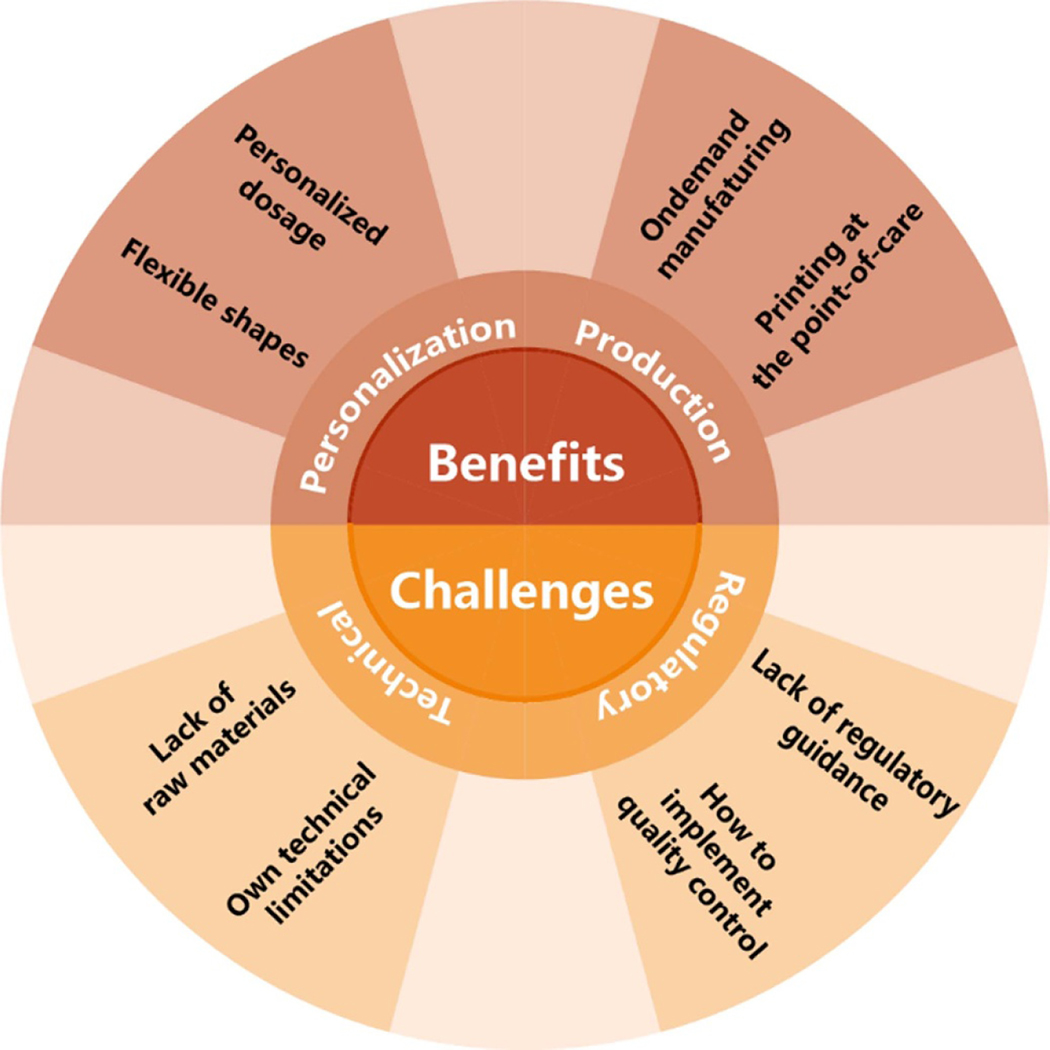
Schematic representation of the benefits and challenges of the 3DP process in drug delivery.

Quality consistency and repeatability are critical factors for successfully applying new manufacturing processes. As a result, most pharmaceutical companies have struggled to scale up the production efficiency of 3DP printing. In addition, many problems, such as technical limitations, high costs of operation and maintenance, and lack of 3DP design knowledge among users, limit the flourishing of the 3DP technology. A 3DP-centric collaboration network and platform can be developed, involving various stakeholders (e.g. equipment and raw material suppliers, educational institutions, technology and service providers, and regulatory authorities) to jointly face challenges, provide solutions, and promote the further development of 3DP in the pharmaceutical industry (figure 13) [258].

## 5. Trends in the future

In the pharmaceutical industry, drug development is a multi-stage process that requires a massive investment of resources and time. Since the 1960s, the industry has been dormant, and progress in the drug manufacturing field has been slow. 3DP offers an opportunity for change in the pharmaceutical industry. It is anticipated that 3DP will serve all the phases of drug development, including drug discovery, clinical trials, drug manufacturing, and first-line medical use [242]. Drugs have a high failure rate in the early stage of development, which would bring a substantial economic burden to the pharmaceutical industry [259]. Therefore, innovative technologies are needed to support drug development, such as rapidly identifying the great drug candidates at a lower cost, producing preparation in a small batch, or even a ‘one-shot’ batch of formulations efficiently, flexibly, and cost-effectively. Because of its unique advantages, 3DP can meet the abovementioned needs and accelerate drug development. In drug discovery, 3DP has already produced APIs [260, 261]. 3DP has also been applied to preclinical drug development, opening new avenues for in vitro pharmacokinetic and toxicological screening experiments. With advances in 3DP technology in the biological field, researchers have been able to build more effective biologically relevant models of tissues and organs of animals and humans by 3DP, thereby improving the speed and accuracy of screening new drug candidates [262]. This will reduce the number of animals needed in the preclinical studies and save development costs of new drugs [263, 264].

For the development of DFs, as highlighted in section 3, 3DP is a vital tool that enables the manufacture of DFs with complex geometric structure, personalized dosage, drug combinations, controlled release or producing DFs of polypills. Compared with traditional manufacturing technologies, 3DP speeds up production, simplifies parameters evaluation such as applicability and compatibility of excipients and drug performance in both in vitro and in vivo models, decreases the development time and cost, and expedites the entry into the phase of clinical trials [265]. Despite the above-mentioned many benefits and enormous development potential of 3DP drugs, the application of this technology has not yet reached the mainstream of clinical practice. Few 3DP drugs have entered clinic assessment, and no drug has followed in the footsteps of Spritam and obtained official approval.

First, the development of 3DP drugs is typically based on trial and error assessments. Here, the flexibility of 3DP is also one of the reasons for its failure. Formula developers have thousands of choices when designing new products, such as the 3DP process, types of drugs and excipients, and fine-tuning printing process parameters. Currently, only a few 3DP technologies and subcategories have been studied in the pharmaceutical field, including material extrusion (FDM and SSE), Vat Photopolymerization (SLA, DLP and CLIP), and the subcategory SLS of Powder bed fusion and Jet Printing (Binder Jetting and Material Jetting). Although these 3DP technologies share some standard features, there are inherent differences in the types of final products they can print. Different 3DP technologies are suitable for producing different personalized drugs due to the advantages and disadvantages of each technology. Their shortages limit some specific applications, and their correlations among these shortages do not overlap in the applications. Therefore, the choice of technology depends on the raw materials used and the final performance of the 3DP product [266]. Each choice must be considered from multiple perspectives, such as formula components, cost, ease of use, maintenance, and regulatory framework. Countless choice criteria often run into trouble, as attempting the entire sample analysis may overwhelm the experiment. In addition, iterative methods for formula development typically consume resources. To realize the commercial feasibility, the decision to develop 3DP drugs should be evidence-based and effective. AI will likely be an essential driver for the clinical translation of 3DP drugs [267]. Machine learning (ML) is a substantial subset of AI that can assist in the formulation screening process of 3DP drugs. ML learns from a large amount of pre-existing data to predict experimental results without considering the number of variables that need to be analyzed. ML can manage 3DP drug formulations in any situation, reducing the demand for professional formula scientists in clinical practice. ML can also direct the preparation process by screening optimized processing parameters, including nozzle diameter, printing temperature, laser intensity, layer thickness, etc [268].

Additionally, integrating 3DP with modern technologies, including intelligent health monitors, Apps, artificial intelligence, and cloud computing, will inaugurate a new era of digital pharmaceuticals. The application of 3DP has been extended from electronics to a broad range of disciplines, including engineering and medicine, where scientific collaboration and interdisciplinary work can help build more brilliant pathways to more reasonable clinical choices [269]. In the future, 3DP may shift drug production from centralized hospitals to decentralized counterparts such as clinics, local pharmacies, or even patients’ homes [233]. Clinicians rely on real-time data online to easily access patients’ electronic medical records to review and modify treatment regimens or dosages. The 3D printer can be controlled remotely, and a doctor or pharmacist can provide the data of printable formulation and send it to a location with a 3D printer for distribution. Experienced patients can even have their 3D printer to dispense medicine, promoting autonomy in the treatment process [270].

**Figure 14.**
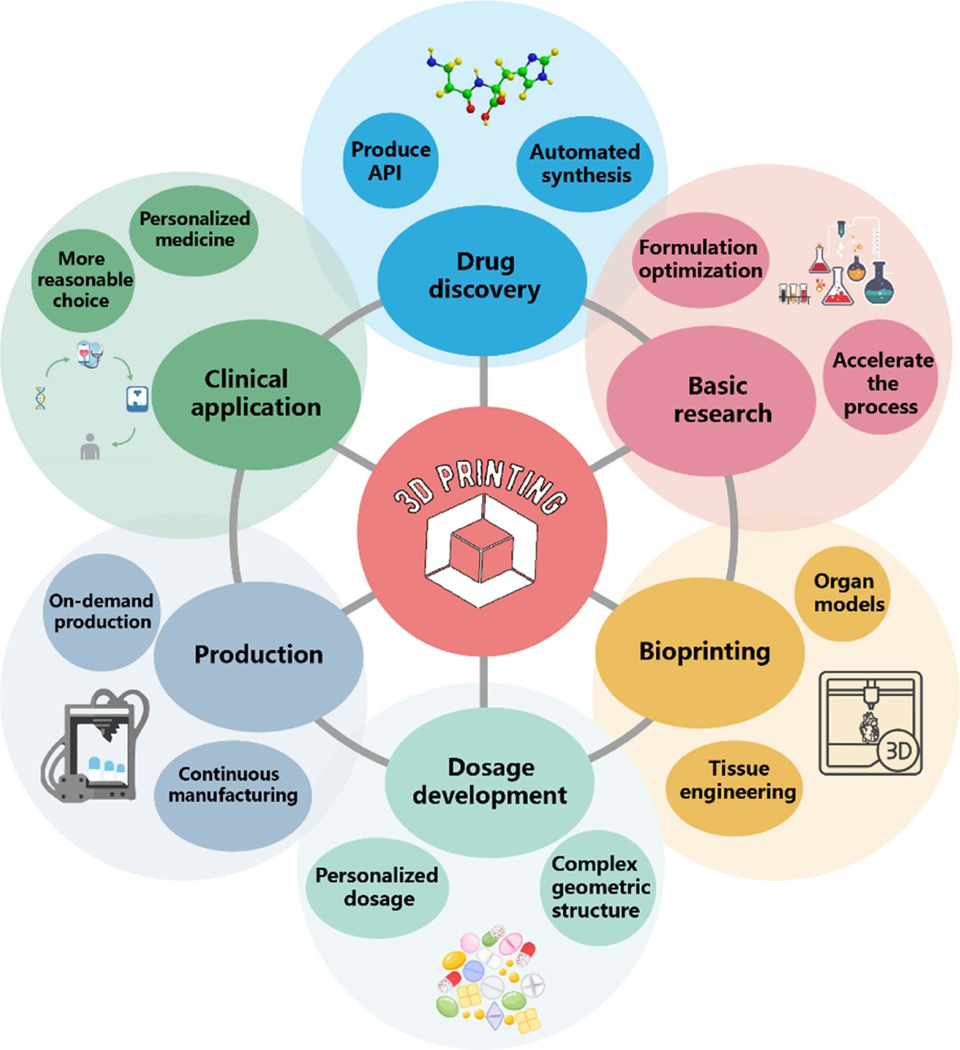
Applications of 3DP in the pharmaceutical fields.

Applying 3DP technology to the pharmaceutical industry will open a new way for personalized medicine, changing how medicines are designed and prescribed for patients and producing novel personalized and programmable medicines. Of course, the development of the 3DP technology is still in the preliminary stage. Some adjustments and advances are needed to turn the technology from conceptual research into practical products and meet people’s needs (figure 14).

## 6. Conclusion

3DP technology enables the DFs to own complex geometric structures, prepare DFs loaded with a few APIs, and regulate multiple release modes to realize personalized DFs’ production on demand. Technology should be increasingly vital in personalized medicine and precise drug delivery. Although 3DP technology has unique advantages with considerable progress, its application in the pharmaceutical industry is still in its infant stage, and it still faces the challenges of quality control, the shortage of polymer excipients, the limitations of its technology, the lack of regulatory regulations, etc. With the rapid increase of 3DP knowledge in the pharmaceutical field and the joint efforts of stakeholders, these problems will eventually be solved. That may mean that we will enter the era of personalized medicine and precise drug delivery once and for all.

## Figures and Tables

**Table 1.** Summary of 3D printing dosage forms, Expected aim, and drugs and excipients used in the literature.

Dosage form	3DP technologies	Drugs/Excipients	Expected aim	References

IR-DFs	Jet printing(JTP)	Clotrimazole and quinapril hydrochloride Ethylcellulose, microcrystalline cellulose, hydroxypropyl methylcellulose, methylcellulose, and polyvinylpyrrolidone	Studies on the formulation of highly porous and fast-disintegrating tablets	[14]
		Amitriptyline hydrochloride Lactose monohydrate, polyvinylpyrrolidone K30 and calcium phosphate dihydrate	Prescription study of immediate-release tablets	[15]
	
	Selective laser sintering (SLS)	5% Acetaminophen and 3% Candurin^®^ Vinyl pyridine-vinyl acetate copolymer (Kollidon VA 64) and hydroxypropyl methylcellulose E5 (HPMC E5)	Oral disintegrating tablets	[16]
		Candurin^®^ NXT, Diclofenac sodium, and Kollidon VA 64 Lactose monohydrate and ruby red	Immediate release of cylindrical tablets	[17]
		Paracetamol, Candurin^®^ Gold Sheen, and Kollidon VA64	Orally disintegrating printouts	[18]
	
	Fused deposition modeling (FDM)	Pantoprazole sodium, PEG 6000, and PVP K12	Immediate release tablets	[19]
		Theophylline, hydroxy propyl cellulose, Eudragit^®^ EPO, Kollidon^®^ VA64, and sodium starch glycolate	Immediate release of the tablets	[20]
		Theophylline, hydroxypropyl cellulose, and triacetin	Immediate release of cellulose tablets	[21]
		Hydrochlorothiazide, triethyl citrate, tri-Calcium phosphate, and Eudragit E	Channelled tablets	[22]
		Theophylline, polyethylene oxides, and PEG 6K	New radiator-shaped tablets	[23]
		Haloperidol, Kollidon^®^ VA64, hydroxypropyl methylcellulose, and glutaric acid	FDM 3DP Immediate release tablets and high drug-polymer ratio	[24]
	
	Semi-solid extrusion (SSE)	Levetiracetam; Polyvinyl alcohol-polyethylene glycol graft copolymer	Tablets	[25]

DR-DFs	Fused deposition modeling (FDM)	Budesonide, polyvinyl alcohol, Eudragit^®^L100, and triethyl citrate Theophylline, polyvinylpyrrolidone, triethyl citrate, and talc	Delayed release tablets	[26]
		Acetaminophen, mannitol, and Eudragit^®^ RS 100	Enteric-coated tablets with delayed release of putamen structure	[27]
			Pressure-controlled delayed-release capsules	[28]
		Oxaliplatin, alginate, calcium chloride, Eudragit L100–55, and PEG 6000	Colon-targeting tablets loaded with nanoparticles	[29]

SR-DFs	Fused deposition modeling (FDM)	Ibuprofen, ethyl cellulose, PVA, PEG6000, Eudragit^®^ RL PO/RS PO, HPMC, and Kollidon^®^ VA64	The role of release regulator in 3D printed tablets	[30]
		Indomethacin, ibuprofen, anhydrous theophylline, polycaprolactone, PEG, and Arabic gum	Polycaprolactone fiber tablets	[31]
		Carvedilol, Affinisol HME 15 LV, HPC SSL, and Eudragit E PO	Applicability of pharmaceutical polymer blends	[32]
		Carbamazepine, triethyl citrate, ethylcellulose, hydroxypropyl cellulose	Zero grade sustained release tablets	[33]
		Theophylline, hydroxypropyl cellulose, polyethylene glycol, and Eudragit^®^ RL PO	Sustained release of theophylline Caplets (PrintCap)	[34]
		Itraconazole, hydroxypropyl cellulose, and polyvinylpyrrolidone	Zero-order sustained-release floating tablets	[35]
		Theophylline, polyvinyl alcohol, hydroxypropyl cellulose, and soluplus	Stomach flotation tablets	[36]
		Cinnarizine, hydroxypropyl cellulose, and Kollidon V A64	Stomach flotation tablets	[37]
		Venlafaxine hydrochloride, hydroxypropyl methylcellulose, and triethyl citrate	Floating sustained-release tablets in the stomach	[38]
		Verapamil hydrochloride, hydroxypropyl methylcellulose, Soluplus^®^, and PEG400	Sustained-release gastric-floating formulation	[39]
		Polyvinyl alcohol filament and domperidone	Gastric retention 3DP device	[40]
	
	Stereolithography (SLA)	4-aminosalicylic acid, paracetamol, polyethylene glycol diacrylate, 2,4,6-trimethyl benzoyl, and PEG 300	Oral modified-release dosage forms	[41]
	
	Digital light processing (DLP)	Paracetamol, PEGDA, 2,4,6-trimethylbenzoyl, PEG400, NaCl, and mannitol	Sustained release tablets	[42]

PR-DFs	Jet printing (JTP)	Fenofibrate, compritol and HD5 ATO	Complex and personalized dosage forms	[43]
	
	Fused deposition modeling (FDM)	Caffeine, hydroxypropyl cellulose, methacrylic acid copolymer, PVA, glycerol, TEC, sodium starch Glycolate and maize starch	Pulsatile-release Chronotopic^™^ tablet	[44]
		Theophylline, Hydroxypropyl cellulose, and ethyl cellulose	Novel core-shell gastro retentive floating pulsatile tablet	[45]
		Acetaminophen, polyvinyl alcohol filament, HPMC, HPMCAS, polyvinyl alcohol-PEG grafted copolymer, PEG, glycerol, and TEC	Multi-compartment capsules for two-pulse oral drug delivery	[46]

Personalized combination and Polypill	Fused deposition modeling (FDM)	Metformin, glimepiride, Eudragit^®^ RL, polyvinyl alcohol, PEG 400, TEC, and PLA	Bilayer structure, oral solid dosage form, a combination of metformin for chronic efficacy and glimepiride for immediate efficacy.	[47]
		Levodopa, benserazide, ethylene-vinyl acetate copolymer, vinylpyrrolidone-vinyl acetate copolymer pramipexole, PVA, and mannitol,	Mini-floating-polypill for Parkinson’s Disease	[48]
		Simvastatin, Aspirin, TEC, Eudragit L100–55, PEG 6000, and PVP k30	A polypill for the prevention of cardiovascular disease	[49]
		Lisinopril dehydrates, amlodipine besylate, indapamide, rosuvastatin calcium, PVA, PLA, and PEG400.	Printing of concept capsules of complex geometry	[50]
	
	Selective laser sintering (SLS)	Paracetamol, ibuprofen, ethyl cellulose N7, Kollicoat, and Candurin Gold Sheen	Small oral dosage forms with modified release features	[51]
	
	Stereolithography (SLA)	Caffeine, paracetamol, naproxen chloramphenicol, aspirin, and prednisolone	A Multi-Layered Polypill Containing Six Drugs	[52]
	
	Jet printing(JTP)	Lisinopril, spironolactone, hydrophilic photocurable bio-ink, and hydrophobic photocurable bio-ink	Combination therapy of hydrophilic and hydrophobic agents in oral dosage forms	[53]

Microneedle	Fused deposition modeling (FDM)	Human VEGF 165, polydimethylsiloxane, 3D-printing resins, triethoxysilane, cefazolin, and NaOH	A wirelessly controlled smart bandage with a 3D-printed miniaturized needle	[54]
		PLA	TDDS with milliprojections	[55]
		Fluorescein, acetone, potassium, and PLA	Biodegradable microneedles	[56]
		Lidocaine hydrochloride ampulla, KOH, methyl red, carmine red, and PLA	Coated 3D printed PLA microneedles	[57]
	
	Stereolithography (SLA)	Rifampicin, biocompatible class I resin, potassium dihydrogen phosphate, disodium hydrogen phosphate, and isopropyl alcohol	3D printed hollow microneedles array	[58]
		UV-curable resin	High-aspect ratio microneedle molds	[59]
		UV-curable resin, sodium carboxymethyl cellulose, sulforhodamine B, and polyvinylpyrrolidone K-30	High-resolution and high-dimensional microneedle mold	[60]
	
	Digital light processing (DLP)	Polyethylene glycol diacrylate, 2,4,6-trimethyl benzoyl	Multi-functional hydrogel microneedles	[61]
	
	Continuous liquid interface production (CLIP)	Rhodamine, trimethylolpropane triacrylate, polyacrylic acid, and photopolymerizable derivatives	Square tapered microneedles	[62]
		EndoGrade endotoxin-free ovalbumin, diphenylphosphine oxide, sucrose, methylcellulose, and sodium alginate	Multilayer microneedles for vaccination	[63]

**Table 2.** Summary of the advantages and disadvantages of various 3DP processes in the pharmaceutical industry in this review.

Additive manufacturing process	Technology	Advantages	Disadvantages	References

Material extrusion	FDM	Operation is simple, and the printing cost is low No solvent involvement No post-processing is required The product has good mechanical properties	Loaded APIs must be resistant to high temperatures The number of thermoplastic polymers available for production is limited Filaments need to be prepared before printing	[85, 247]
	
	SSE	Low-temperature exposure cheap, easy to use and readily available multi-material printing	Limited resolution; requires post-processing low mechanical properties	[77]

Vat Photopolymerization	SLA	High precision and accuracy	Potential toxicity Requires post-processing and support Mechanical properties decrease over time	[248]
	
	DLP	Higher resolution and smooth finish for small objects	Requires support Mechanical properties decrease over time Restricted by pixel size Potential toxicity	[248]
	
	CLIP	Fastest 3D printing technology; high precision	Potential toxicity Equipment is relatively expensive	[129]

Powder bed fusion	SLS	Solvent-free process It is faster than other technologies	High temperature and high energy lasers may affect the stability of drugs and polymers	[135, 139]
		No post-processing To form an ideal IR dosage form with high porosity	The recycling/reuse of raw materials will have an impact on the quality of products	
			The mechanical features and surface roughness of the products are poor	

Binder jetting	BJ	No need for support Multimaterial printing	Poor mechanical strength and friability Post-treatment drying is required Low selection of materials	[142, 144]

Material jetting	MJ	Multi-material printing High accuracy Ecofriendly	Low mechanical properties; Costly Support needed	[249]

## Data Availability

All data that support the findings of this study are included within the article (and any supplementary files).
